# NanoBind: Mechanism-Driven Deep Learning of Nanobody–Antigen Molecular Recognition

**DOI:** 10.34133/research.1327

**Published:** 2026-06-23

**Authors:** Shiqing Zhao, Yanhao Zhu, Ruizhou Li, Zeyu Xu, Mingming Han, Jiyun Han, Qiuyu Li, Mingming Guan, Likun Wang, Juntao Liu, Lijie Xing

**Affiliations:** ^1^ School of Mathematics and Statistics, Shandong University, Weihai 264209, China.; ^2^ Department of Lymphoma, Shandong Cancer Hospital and Institute, Shandong First Medical University and Shandong Academy of Medical Sciences, Jinan 250117, China.; ^3^ Institute of Systems Biomedicine, School of Basic Medical Sciences, Peking University Health Science Center, Beijing 100191, China.; ^4^ Department of Medical Genetics, School of Basic Medical Sciences, Peking University Health Science Center, Beijing 100191, China.

## Abstract

Nanobody–antigen molecular recognition underpins nanobody discovery and development, necessitating accurate determination of binding occurrence, interface residues, and affinity. Current predictors are architecturally designed for the massive, heterogeneous spectrum of general protein–protein interactions, diluting the limited, complementarity-determining region (CDR)-dominated nanobody–antigen interaction (NAI) data and masking the decisive CDR signal. The scarcity of experimental affinity data precludes direct regression-based estimation of binding affinity. Here, we present NanoBind, a mechanism-driven deep learning framework that embeds the CDR-dominated binding pattern within its encoder, enabling robust prediction of binding occurrence and interface residues from limited NAI data. Constrained by scarce affinity data, NanoBind generates quantitative affinity ranges for nanobody–antigen pairs without extra experiments. Systematic benchmarking demonstrates that NanoBind surpasses state-of-the-art methods in accuracy and robustness, and interpretability analyses confirm that the model’s decisions align with the CDR-dominated binding mechanism. When million-sequence immune repertoires are screened against 4 antigens, NanoBind reduces candidate nanobodies to fewer than 100 per target. For the severe acute respiratory syndrome coronavirus 2 (SARS-CoV-2) spike receptor-binding domain (RBD)–nanobody F2 complex, NanoBind correctly predicts binding occurrence, matches experimentally validated interface residues, and generates an affinity range quantitatively supported by molecular dynamics simulations. A server is available at http://liulab.top/NanoBind/server.

## Introduction

**Fig. 1.**
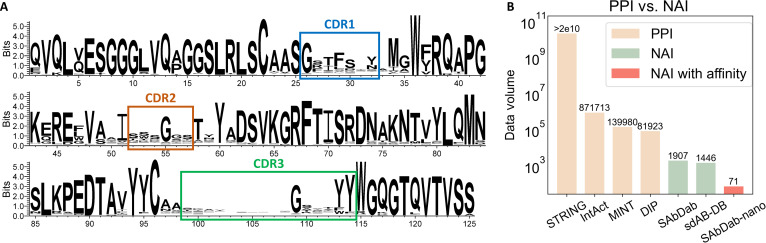
Sequence conservation in nanobodies and the scale of existing datasets for PPIs and NAIs. (A) Sequence logo showing amino acid frequencies in 10,000 nanobody sequences randomly sampled from the INDI database [55]. The 3 hypervariable CDRs, particularly CDR3, are highlighted with colored boxes. (B) Data volume comparison of mainstream PPI and NAI databases. Publicly available NAI datasets are substantially smaller than PPI datasets, with an especially pronounced gap for NAIs with experimentally validated binding affinities.

Nanobodies are single-domain antibodies [1] that achieve precise antigen recognition through 3 hypervariable complementarity-determining regions (CDRs; Fig. 1A) [2–4]. Because of their small size [5], high stability [6], and low immunogenicity [7], they have expanded in diagnostics [8,9], oncology [10], and antiviral therapy [11,12]. Understanding nanobody–antigen molecular recognition underpins clinical discovery and development, necessitating answers to 3 key questions: Does binding occur? Where does binding occur? How strong is the binding? Binding occurrence supports hit identification [13]; interface mapping guides residue-level nanobody optimization; affinity estimation enables selection of developable leads [14].

Existing approaches for determining molecular recognition can be categorized into experimental and computational methods. Experimental techniques [15–18] such as surface plasmon resonance (SPR) [15] and x-ray crystallography [17] accurately determine occurrence, interface residues, and affinities. However, these methods are costly and time-consuming, making large-scale analysis of nanobody–antigen molecular recognition impractical. Computational approaches such as molecular docking [19] and all-atom molecular dynamics (MD) [20,21] simulations provide atomic-level insights, but they depend on high-resolution structures of both partners [22] and require tremendous computational resources.

With the advancement of deep learning [23–27], numerous models for protein–protein interactions (PPIs) [28–33], interface residues [34–39], and affinity [40–42] prediction have been developed, enabling large-scale protein–protein recognition. However, these predictors perform poorly in nanobody–antigen recognition prediction [43] for 2 reasons. First, they are trained on large, heterogeneous datasets (Fig. 1B) that statistically dilute the limited CDR-dominated nanobody–antigen interaction (NAI) [44] data, masking the decisive CDR signals [44]. Secondly, their architectures are tuned to broad, global interaction patterns of general PPIs and therefore fail to capture the compact, local CDR-dominated binding pattern underlying nanobody–antigen recognition. In binding prediction, current models such as D-SCRIPT [45], Topsy-Turvy [46], and PIPR [47] infer interactions by generating whole-chain contact maps or aggregating global representations, which dilute the critical signals derived from local CDR loops [48]. In interface residue prediction, existing models such as SCRIBER [49], ScanNet [50], and MaSIF-site [51] identify only generic, partner-agnostic surface patches, failing to characterize the complementarity between specific nanobody–antigen pairs and to localize the true interface. In affinity prediction, models such as PIPR, ANTIPASTI [41], and PPA-Pred [42] either model broad interaction interfaces through global encoders that obscure local CDR contributions or are explicitly designed for 2-chain antibodies. Despite recent progress, nanobody-specific models still under-exploit CDR-dominated binding mechanisms, and the severe scarcity of affinity data (Fig. 1B) renders precise, regression-based affinity prediction currently infeasible [52]. DeepNano [53], the first dedicated model, leverages embeddings from a protein language model and designs a prompt encoder to focus on binding interfaces, yet its predictive power is still constrained by the limited NAI data because it does not explicitly model CDR-dominated binding mechanisms.

Here, we present NanoBind, a mechanism-driven deep learning framework for comprehensive prediction of nanobody–antigen molecular recognition. At its core, NanoBind encodes CDR-centric binding rules through complementary global and local views: The Global Adaptive Module captures long-range dependencies and relative spatial positioning between CDRs via rotary positional embedding, while the Local Adaptive Module extracts short-range motifs and residue-level patterns within CDRs through a small-kernel one-dimensional convolutional neural network (1D-CNN). This architecture explicitly embeds the defining characteristic of NAIs, CDR-dominated binding recognition, rather than relying on implicit learning from heterogeneous data, enabling robust performance under data-limited conditions.

Building on this encoder, NanoBind deploys 5 integrated submodels that collectively deliver a complete profiling pipeline: (a) NanoBind-seq predicts binding occurrence by integrating nanobody–antigen co-activation features; (b) NanoBind-site locates antigen-binding interfaces with nanobody-guided cross-attention; (c) NanoBind-pro enhances binding prediction by incorporating predicted interface residues as structural prompts; (d) NanoBind-pair performs pairwise affinity comparisons between 2 complexes, serving as a standalone tool for relative affinity ranking; and (e) NanoBind-affi estimates affinity ranges by leveraging NanoBind-pair to compare target complexes against 49 reference complexes spanning 10^−12^ to 10^−4^ M, circumventing the data scarcity that precludes direct regression. Collectively, these integrated submodels enable NanoBind to deliver a complete pipeline for profiling nanobody–antigen molecular recognition.

Comprehensive benchmarking shows that NanoBind surpasses state-of-the-art accuracy across all 3 tasks, while interpretability analyses through ablation studies, masking experiments, and attention visualization confirm that its decisions are mechanistically grounded in CDR-dominated recognition rather than spurious correlations. On the severe acute respiratory syndrome coronavirus 2 (SARS-CoV-2) spike receptor-binding domain (RBD)–nanobody F2 complex [Protein Data Bank (PDB): 7OAY], NanoBind correctly predicts binding, fully recapitulates experimentally validated interface residues [54], and yields an affinity range consistent with MD simulations and close to the experimental value, illustrating its end-to-end application pipeline. Finally, virtual screening against 4 antigens [glutathione *S*-transferase (GST), lysozyme, pfVAR2CSA, and PD-L1] demonstrates NanoBind’s ability to enrich true binders from million-scale libraries to fewer than 100 candidates per target for experimental prioritization. A user-friendly web server (http://liulab.top/NanoBind/server) provides immediate access to all prediction modules.

## Results

### Overview of NanoBind framework

**Fig. 2.**
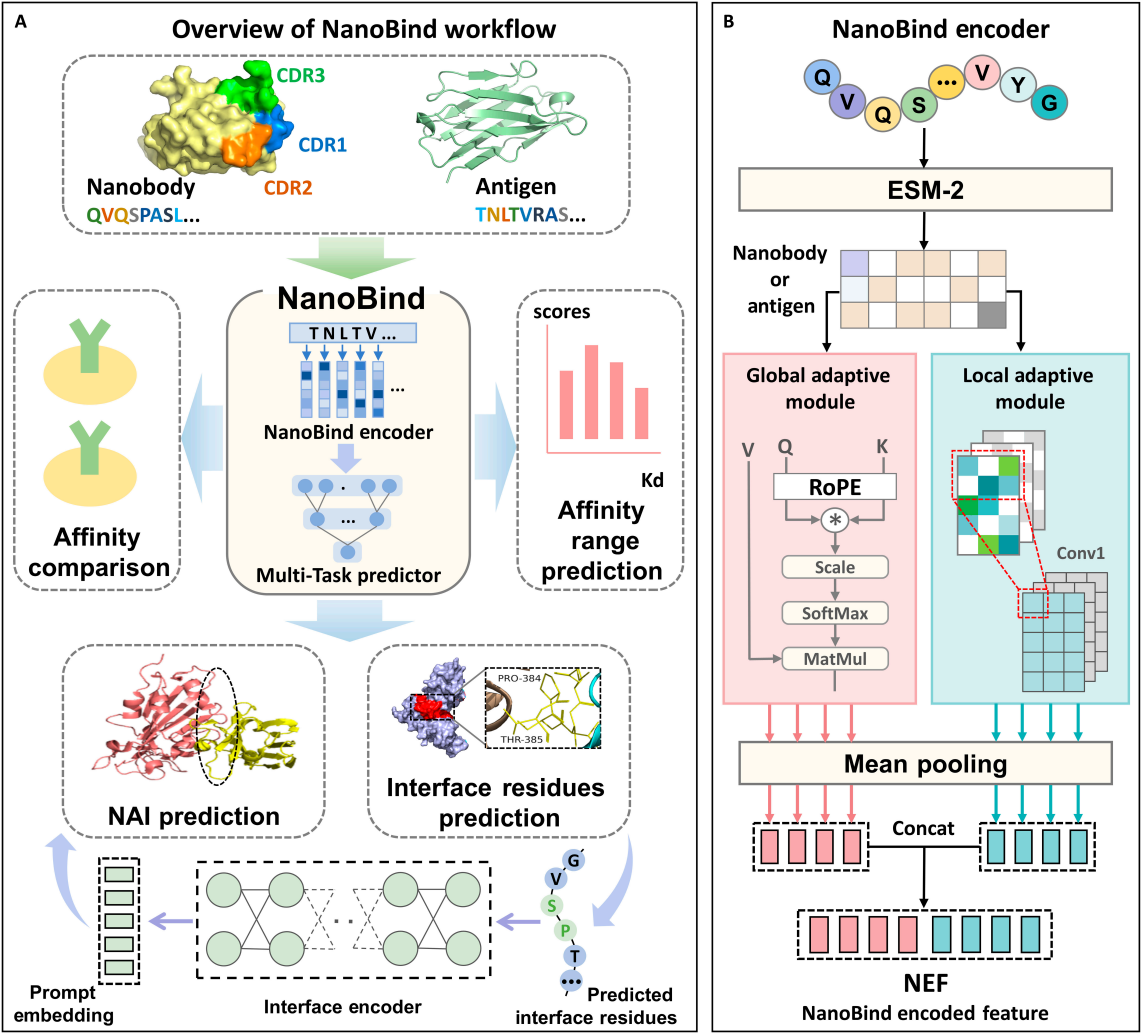
The NanoBind framework. (A) Overview. NanoBind offers a unified framework for nanobody–antigen molecular recognition. It utilizes a shared NanoBind Encoder to extract features from nanobodies and antigens, enabling systematic prediction of binding, interface residues, relative affinity, and affinity ranges. (B) Encoder architecture. For an input amino acid sequence, the NanoBind Encoder first generates residue-level embeddings using ESM-2. Subsequently, the Global Adaptive Module [rotary positional embedding (RoPE) [56]-enhanced self-attention] captures long-range CDR cooperativity, while the Local Adaptive Module (1D convolution) extracts local patterns specific to the CDRs. After global average pooling, the 2 feature streams are concatenated to form the NanoBind Encoder Feature (NEF).

As shown in Fig. 2A, NanoBind is a unified, mechanism-driven deep learning framework for predicting nanobody–antigen molecular recognition from sequences. It embeds the CDR-dominated binding mechanism within a shared encoder. Task-specific predictors built on this encoder enable accurate predictions of binding occurrence, interface residues, and affinity range.

**Fig. 3.**
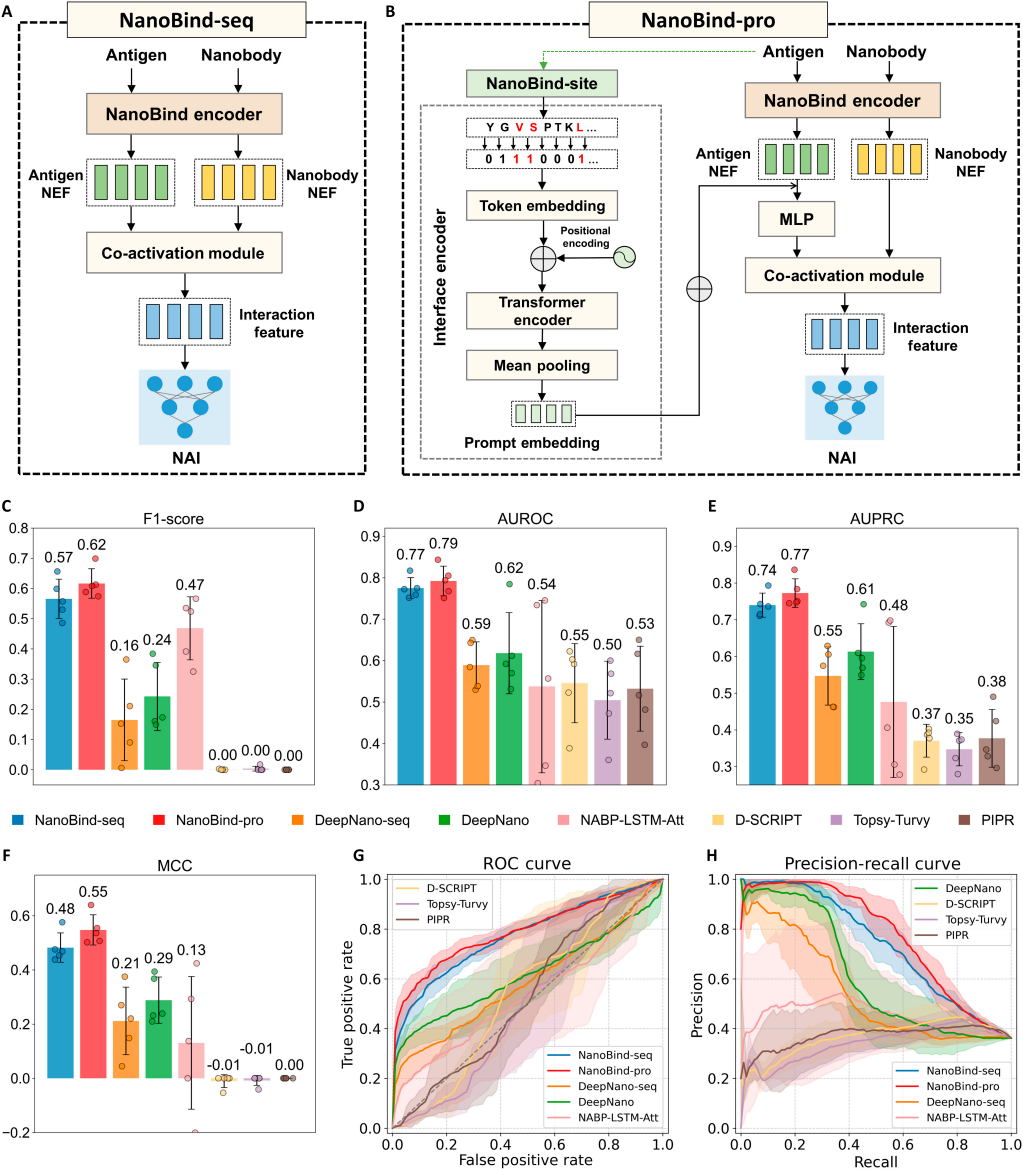
Overview of NanoBind-seq and NanoBind-pro and their performance in NAI prediction. (A) NanoBind-seq framework for predicting NAIs from nanobody and antigen sequences. (B) NanoBind-pro framework, enhanced with an Interface Encoder to incorporate the binding interface information for NAI prediction. (C to F) Quantitative comparison of NanoBind-seq and NanoBind-pro against 5 baselines across F1-score, MCC, AUROC, and AUPRC. Bars show mean scores (5 independent runs); black error bars and scatter points indicate standard deviation and individual run scores. (G and H) Receiver operating characteristic (ROC) and precision-recall (PRC) curves contrasting NanoBind-seq, NanoBind-pro, and baselines. Solid lines and shaded areas, respectively, represent mean and standard deviation of 5 runs.

**Fig. 4.**
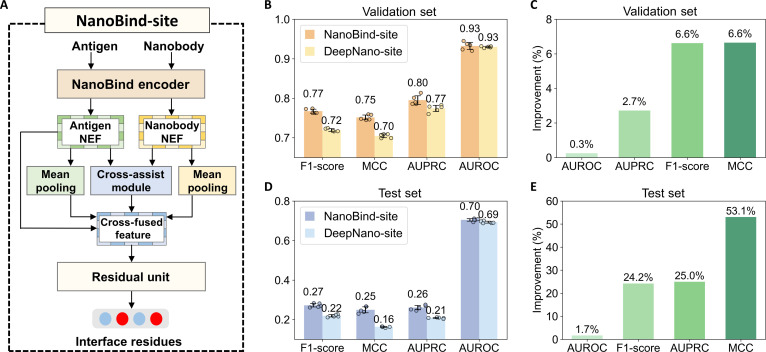
Overview of NanoBind-site and its performance in interface residue prediction. (A) Framework of NanoBind-site for predicting nanobody-specific antigen-interface residues using features from both nanobodies and antigens. (B) Comparison of NanoBind-site against DeepNano-site on the validation set (F1-score, MCC, AUPRC, AUROC). Bars show mean scores from 5 runs; black error bars and scattered points indicate SD and individual scores. (C) Relative improvement of NanoBind-site over DeepNano-site on the validation set. (D) Comparison of NanoBind-site against DeepNano-site on the independent test set [metrics as in (B)]. (E) Relative improvement of NanoBind-site over DeepNano-site on the test set.

**Fig. 5.**
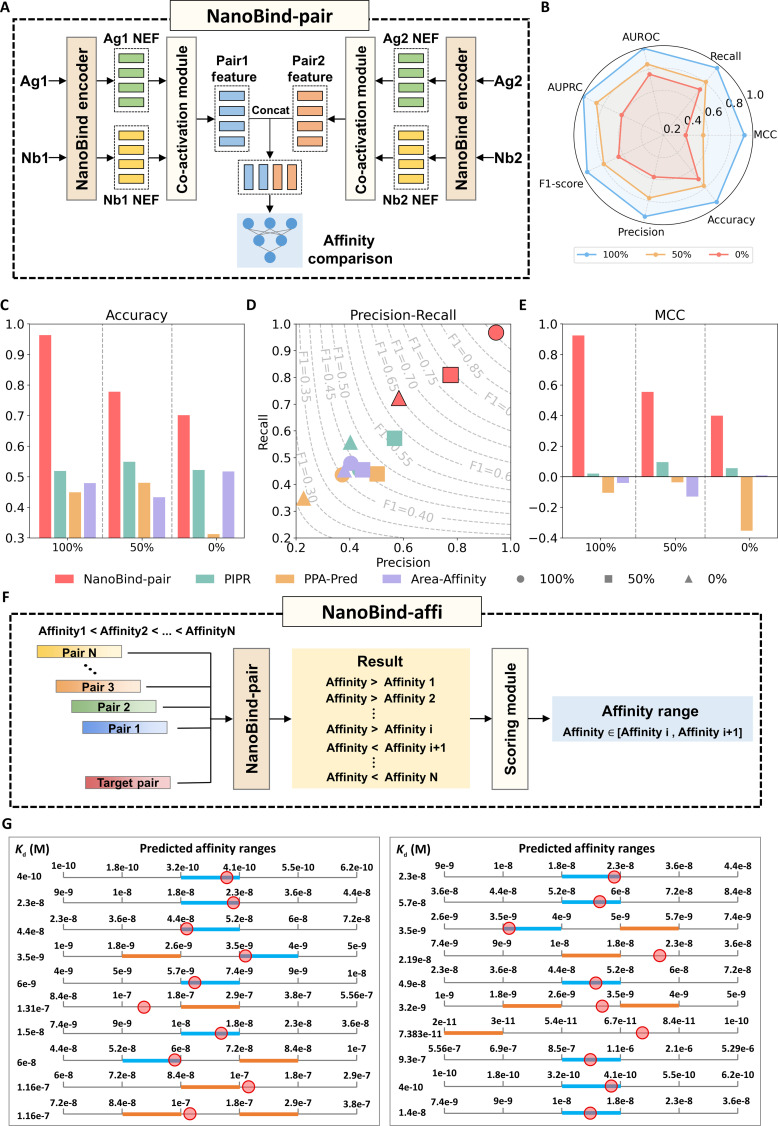
Overview of NanoBind-pair and NanoBind-affi, and their performance in affinity prediction. (A) NanoBind-pair framework for relative affinity comparison. (B) Performance of NanoBind-pair under 100%, 50%, and 0% dataset overlap. (C to E) Comparison with 3 baselines across metrics (ACC, F1-score, Recall, Precision, MCC). (F) NanoBind-affi framework for affinity range estimation via NanoBind-pair. (G) Affinity range prediction for 20 complexes. Red dots indicate actual *K*_d_ values; blue and orange bands respectively represent correct and incorrect predictions.

Given amino acid sequences of a nanobody and its target antigen, the NanoBind Encoder (Fig. 2B) first generates residue-level embeddings via ESM-2. A Global Adaptive Module then captures long-range dependencies and relative positional relationships between CDRs, while a Local Adaptive Module extracts local sequence patterns and short-range motifs within CDRs. After global average pooling, the 2 pathway outputs are concatenated to form the NanoBind Encoder Feature (NEF), encoding CDR-dominated binding signatures. Based on these NEFs, NanoBind deploys several task-specific predictors. NanoBind-seq (Fig. 3A) employs a Co-Activation Module to generate interaction features and predict binding probability. NanoBind-site (Fig. 4A) uses a Cross-Assist Module to extract nanobody-guided features and localize antigen-interface residues. NanoBind-pro (Fig. 3B) incorporates predicted interface residues via an Interface Encoder to refine NAI prediction. For affinity estimation under scarce data, NanoBind-pair (Fig. 5A) employs the Co-Activation Module to compare relative affinity strengths between complexes. NanoBind-affi (Fig. 5F) benchmarks the query complex against references of known affinity via NanoBind-pair and then integrates the scores to determine the most probable affinity range.

### Hierarchical architecture of nanobody–antigen molecular recognition

NanoBind profiles molecular recognition through 3 hierarchical tiers: binding occurrence (Tier 1), interface localization (Tier 2), and affinity estimation (Tier 3). Tier 1 comprises NanoBind-seq and NanoBind-pro, with the latter incorporating Tier 2 interface predictions as structural prompts to refine binding assessment. Tier 2 (NanoBind-site) localizes antigen-interface residues via nanobody-guided cross-attention. Tier 3 (NanoBind-affi) estimates affinity ranges by leveraging NanoBind-pair (a dedicated pairwise comparison engine) to benchmark target complexes against 49 reference complexes, circumventing the data scarcity that precludes direct regression. The following sections present each component in sequence, with emphasis on how information flows between tiers to create an integrated pipeline.

### NanoBind-seq enables accurate and robust NAI prediction

To systematically evaluate the ability of NanoBind-seq (Fig. 3A) to predict NAIs, we trained it on the NAI dataset (1,019 positive and 10,190 negative pairs) used by DeepNano [53] and tested it on the independent test set (651 positive and 1,149 negative pairs) from the sdAb-DB [57] dataset (detailed in Methods). We benchmarked it against existing NAI predictors (DeepNano-seq [53], DeepNano, and NABP-LSTM-Att [58]) and 3 leading general PPI models (D-SCRIPT [45], Topsy-Turvy [46], and PIPR [47]), with 5 replicated training sessions on the identical dataset (parameter settings in Note S1). Due to class imbalance, accuracy (ACC) offers limited reference. Therefore, we selected area under the receiver operating characteristic curve (AUROC), area under the precision-recall curve (AUPRC), F1-score, and Matthews correlation coefficient (MCC) to assess ranking ability and balanced discrimination. Detailed results are provided in Table 1 and Table S1, while the calculation methods for the evaluation metrics are described in Notes S2 and S3.

**Table 1.** Performance of NanoBind-seq, NanoBind-pro, and other compared methods on NAI prediction

Method	MCC	F1-score	AUROC	AUPRC
DeepNano-seq	0.212 ± 0.125	0.165 ± 0.135	0.589 ± 0.056	0.547 ± 0.079
DeepNano	0.288 ± 0.085	0.243 ± 0.113	0.618 ± 0.098	0.613 ± 0.076
NABP-LSTM-Att	0.131 ± 0.245	0.468 ± 0.104	0.537 ± 0.208	0.476 ± 0.206
D-SCRIPT	−0.011 ± 0.024	0.001 ± 0.001	0.546 ± 0.096	0.370 ± 0.045
Topsy-Turvy	−0.008 ± 0.018	0.004 ± 0.008	0.504 ± 0.094	0.347 ± 0.045
PIPR	0 ± 0.000	0 ± 0.000	0.532 ± 0.103	0.377 ± 0.079
NanoBind-seq	0.482 ± 0.054	0.566 ± 0.065	0.775 ± 0.025	0.740 ± 0.033
NanoBind-pro	0.547 ± 0.056	0.616 ± 0.049	0.792 ± 0.036	0.773 ± 0.039

As shown in Fig. 3C to H, NanoBind-seq consistently outperformed all baselines across 5 runs, achieving higher average scores and lower variance in all 4 metrics. General PPI models (D-SCRIPT, Topsy-Turvy, PIPR) collapsed despite retraining, yielding near-zero recall, F1-scores (0.000 to 0.004), and MCC values (−0.01 to 0.00) indistinguishable from random guessing, highlighting that architectures tuned to large, distributed PPI interfaces cannot detect highly localized CDR signals under data-limited NAI conditions. DeepNano achieved AUROC 0.62 ± 0.10, AUPRC 0.61 ± 0.08, F1-score 0.24 ± 0.11, and MCC 0.29 ± 0.09, yet still misclassified most true binders. NanoBind-seq, built for CDR-dominated recognition, reached AUROC 0.78 ± 0.03, AUPRC 0.74 ± 0.03, F1-score 0.57 ± 0.07, and MCC 0.48 ± 0.05, representing relative gains of 25.4%, 20.7%, 133.2%, and 67.3% over DeepNano, thus establishing a new benchmark for NAI prediction.

### NanoBind-site achieves accurate and robust antigen-interface residue prediction

Following Tier 1 binding prediction, Tier 2 interface localization identifies where binding occurs. To evaluate NanoBind-site (Fig. 4A) under nanobody-specific conditions, we assessed its performance on the SAbDab-nano [59] dataset, a subset of the Structural Antibody Database [60] (detailed in Methods). Owing to the limited generalization of general PPI models in NAI field, only DeepNano-site [53], which targets nanobody–antigen binding residue prediction, was used for comparison (parameter settings in Note S1). Residue-level interface identification is highly imbalanced (≤30% of interface residues on average [53]), so F1-score, MCC, AUPRC, and AUROC were used to assess model performance under imbalance. Both models were evaluated in 5 independent runs with different random seeds.

As shown in Fig. 4B to E, NanoBind-site exhibited higher accuracy and greater stability than DeepNano-site on both validation and test sets (detailed in Table 2 and Table S2). On the validation set, it improved F1-score from 0.72 to 0.77, MCC from 0.70 to 0.75, and AUPRC from 0.77 to 0.80. On the test set, NanoBind-site achieved relative gains of 53.1% in MCC, 25.0% in AUPRC, 24.2% in F1-score, and 1.7% in AUROC. These consistent improvements demonstrate that NanoBind-site reliably discriminates true antigen-interface residues under nanobody-specific conditions.

**Table 2.** Performance of NanoBind-site and other compared method in the test set

Method	MCC	F1-score	AUROC	AUPRC
DeepNano-site	0.163 ± 0.003	0.219 ± 0.007	0.693 ± 0.006	0.208 ± 0.003
NanoBind-site	0.249 ± 0.017	0.272 ± 0.010	0.705 ± 0.007	0.260 ± 0.012

### NanoBind-pro enhances NAI prediction by incorporating predicted interface residues

Building upon Tier 2 interface predictions, NanoBind-pro incorporates predicted binding residues as structural prompts to refine Tier 1 binding occurrence prediction. Although NanoBind-seq achieves robust NAI prediction by focusing on nanobody CDR-dominated signals, it still treats all antigen residues equally, diluting the contribution of the small but decisive interface residues [61]. Prior knowledge of these limited interface residues, therefore, provides complementary structural cues for more accurate and physically grounded NAI prediction. To this end, we integrated the antigen-interface residues predicted by NanoBind-site as prompts into NanoBind-seq through an Interface Encoder, constructing NanoBind-pro (Fig. 3B). As shown in Fig. 3C to H, trained and tested on the same dataset, NanoBind-pro improved over NanoBind-seq by 8.8%, 14.6%, 2.6%, and 4.05% in F1-score, MCC, AUROC, and AUPRC, respectively (detailed in Table 1 and Table S1), demonstrating that jointly considering nanobody CDRs and the binding interface enhances NAI prediction.

### NanoBind-affi accurately estimates affinity ranges under data scarcity

At Tier 3, NanoBind-affi estimates affinity ranges by leveraging NanoBind-pair to benchmark target complexes against reference anchors, circumventing the data scarcity that precludes direct regression. For affinity estimation, only 185 experimentally annotated nanobody–antigen complexes with dissociation constant (*K*_d_) values were available from SAbDab-nano and relevant literature (detailed in Note S4). First, all pairwise comparisons were generated to train the affinity comparison model NanoBind-pair (Fig. 5A). Next, 49 complexes with distinct *K*_d_ values were selected as a reference set, defining 50 contiguous affinity ranges (selection criteria in Note S4). We then constructed NanoBind-affi, which utilizes NanoBind-pair to sequentially compare the target complex against these reference anchors. Through an interval scoring module, it locates a specific interval among the 50 ranges as the estimated affinity range of the target (Fig. 5F). To evaluate NanoBind-pair, we constructed 3 test sets with 100%, 50%, and 0% overlap with the training set (detailed in Methods) and compared against 3 regression-based PPI affinity predictors (PIPR, PPA-Pred [42], and Area-Affinity), whose predictions were converted into relative rankings (parameter settings in Note S1). Evaluation metrics included ACC, Precision, Recall, F1-score, MCC, AUROC, and AUPRC. Furthermore, we evaluated NanoBind-pair in terms of overfitting, homology, and generalization by partitioning the datasets according to antigen sequence identity (detailed in Note S5 and Tables S7 and S8), and it demonstrates a reliable generalization under strictly nonhomologous conditions without overfitting.

As shown in Fig. 5C to E, NanoBind-pair achieved excellent performance across all 3 test sets. On the 100% overlap test set, it attained ACC 0.96, MCC 0.92, Recall 0.97, Precision 0.95, and F1-score 0.96. Even on the 0% overlap test set, ACC remained 0.70 and Recall 0.72. In contrast, baseline models performed near-randomly (ACC ≈ 50%, MCC ≈ 0; detailed in Tables S3 to S5), demonstrating that architectures tuned to general PPIs fail to capture nanobody–antigen specificity. To evaluate NanoBind-affi, we randomly selected 20 complexes from 136 available complexes, ensuring no pairwise overlap with the 49 reference complexes. As shown in Fig. 5G, 14 ranges were correct, 5 fell within adjacent bins, and only one spanned 3 bins with minimal discrepancy (~10^−11^ M). To exclude potential anchor-selection bias, we randomly substituted 20% and 40% of the reference anchors. NanoBind-affi robustly maintained a cumulative match rate (predicting the exact or adjacent interval) of 0.97 ± 0.03 and 0.95 ± 0.05, respectively (Fig. S1). These results demonstrate that the interval estimation is highly stable to anchor perturbation. Thus, even under data scarcity, NanoBind-affi provides accurate affinity range estimates.

### NanoBind accurately characterizes molecular recognition for the nanobody F2–RBD complex

**Fig. 6.**
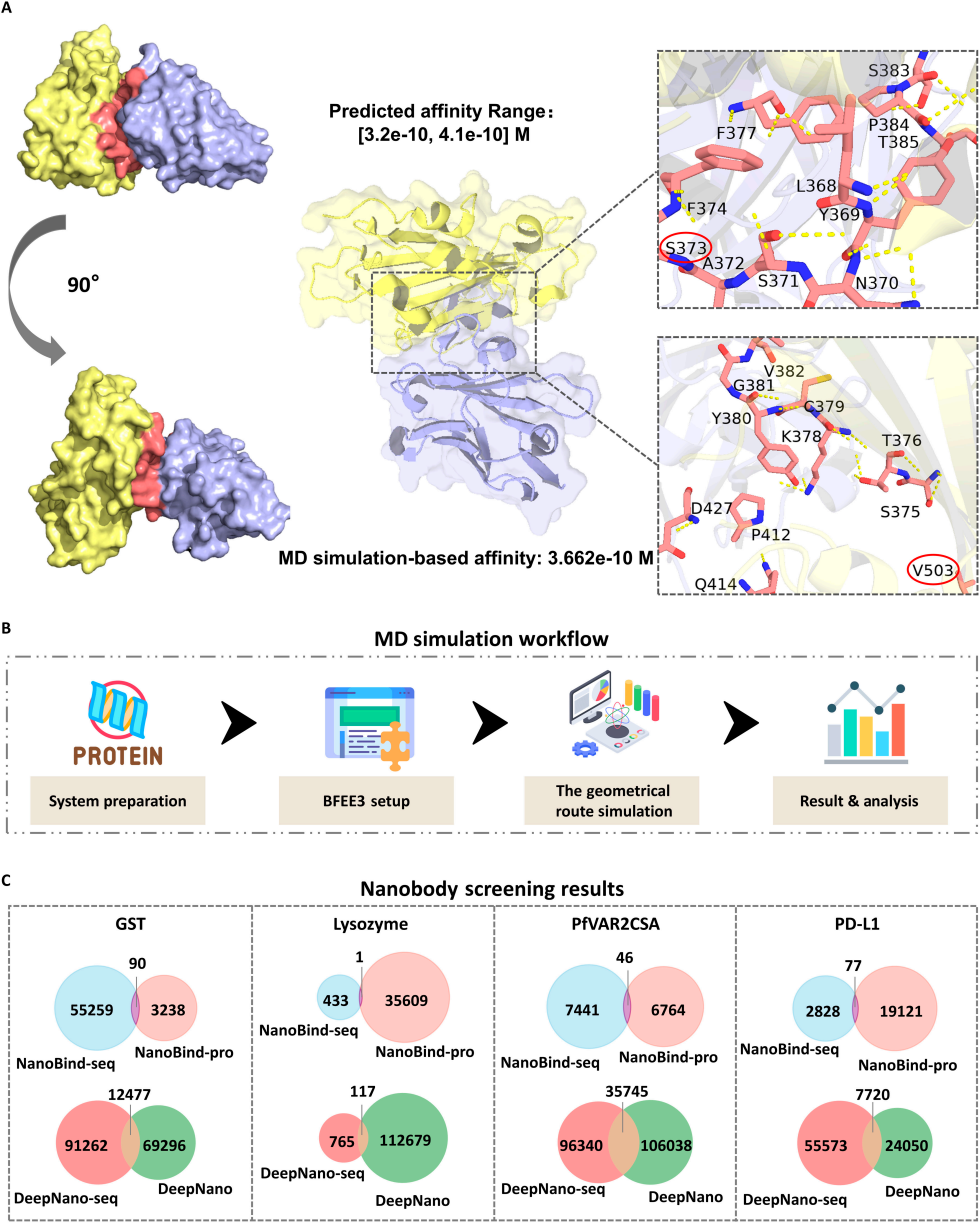
NanoBind predictions for the nanobody F2–RBD complex (7OAY) and screening results. (A) Interface residue visualization. Left, 7OAY structure; right, 22 residues predicted by NanoBind-site with 20 experimentally validated (S373 and V503 circled, unconfirmed). (B) MD simulation workflow. (C) Venn diagram of candidates prioritized by NanoBind-seq, NanoBind-pro, DeepNano-seq, and DeepNano in million-scale screening against 4 antigens. Overlaps indicate joint predictions.

To demonstrate the integrated Tier 1→Tier 2→Tier 3 pipeline and NanoBind's predictive behavior in a concrete biological context, we applied NanoBind to the well-characterized SARS-CoV-2 RBD–nanobody F2 complex (PDB: 7OAY), which is a major target for neutralizing nanobodies. Nanobody F2 binds RBD (PDB: 7OAY) and sterically blocks ACE2 engagement [62], preventing viral entry (detailed in Note S6). Specifically, we first used NanoBind-seq and NanoBind-pro to predict the binding occurrence for the 7OAY complex; both correctly identified the binding. Upon this positive prediction, we then applied NanoBind-site to predict the antigen-interface residues, and NanoBind-affi to estimate the affinity range. Interface predictions (Fig. 6A) showed high overlap with experimental data. Twenty of the 22 predicted binding residues were experimentally supported, including L368-A372, F374-T385, P412, Q414, and D427, encompassing key escape mutation sites such as Y369H, S371P, F377L, and K378Q/N [54]. Two high-scoring predictions, V503 and S373, lack direct contact evidence but are mechanistically plausible (detailed in Note S6).

NanoBind-affi predicted an affinity range of [3.2 × 10^−10^, 4.1 × 10^−10^] M, close to the experimental value of 4 × 10^−11^ M. To further validate this, we performed MD simulations using CHARMM-GUI and the BFEE3 constrained sampling protocol, gradually releasing spatial restraints on the ligand (Fig. 6B; detailed in Fig. S2 and Note S7). The calculated binding free energy corresponded to *K*_d_ 3.662 × 10^−10^ M, falling within our predicted range. This consistency indicates that NanoBind-affi achieves MD-level precision from sequence alone at a far lower computational cost.

### Screening target nanobodies from a 1 million natural nanobodies library

Beyond single-complex characterization, NanoBind enables efficient high-throughput identification of antigen-specific nanobodies from large-scale natural sequence libraries. High-throughput discovery of antigen-specific nanobodies relies heavily on the rapid and accurate prioritization of candidates from massive sequence libraries. To evaluate practical screening utility, we collected 4 target antigens—GST, lysozyme, *Plasmodium falciparum* VAR2CSA (pfVAR2CSA), and human programmed death-ligand 1 (PD-L1)—along with their corresponding experimentally verified binding nanobodies and a background library of 1 million natural nanobody sequences (detailed in Methods). We employed NanoBind-seq, NanoBind-pro, DeepNano-seq, and DeepNano to predict binding probabilities between the million-scale nanobody library and the target antigens, and subsequently ranked the candidates based on these predictions. Performance was measured by the rank of the true binding nanobodies among the massive background; a higher ranking (i.e., a smaller rank index) indicates that substantially less experimental screening is required to successfully identify at least one true binder.

As shown in Table 3, NanoBind-seq and NanoBind-pro consistently identified true binders at significantly higher ranks compared to DeepNano-seq and DeepNano. In the lysozyme antigen screening, following the rankings provided by NanoBind-seq requires only 434 top-ranked candidates to be experimentally tested to guarantee the discovery of at least one true binder. Furthermore, given the high precision demonstrated by the NanoBind models in the benchmarking tests, we deduce that undiscovered true binders highly likely exist among the top-scoring candidates preceding the known binders. By extracting these top-scoring candidates and taking the intersection of the predictions from both NanoBind-seq and NanoBind-pro, we further narrowed down the candidate pool (Fig. 6C). This intersection provides a high-confidence prioritized set of nanobody candidates for future experimental screening (detailed intersecting nanobody sequences are provided in Tables S9 to S12, and precision validation for the simultaneous use of NanoBind-seq and NanoBind-pro is detailed in Note S8).

**Table 3.** The rank of the highest-scoring known binder predicted by NanoBind-seq, NanoBind-pro, DeepNano-seq, and DeepNano

Method	The rank of the highest-scoring known binder			
	GST	Lysozyme	pfVAR2CSA	PD-L1
NanoBind-seq	55,260	434	7,442	2,829
NanoBind-pro	3,239	35,610	6,765	19,122
DeepNano-seq	91,263	766	96,341	55,574
DeepNano	69,297	112,680	106,039	24,051

### Ablation studies on NanoBind

To systematically evaluate the contribution of each component to NanoBind, we conducted ablation experiments on NanoBind-seq, NanoBind-site, and NanoBind-pro by sequentially removing or replacing individual modules.

**Fig. 7.**
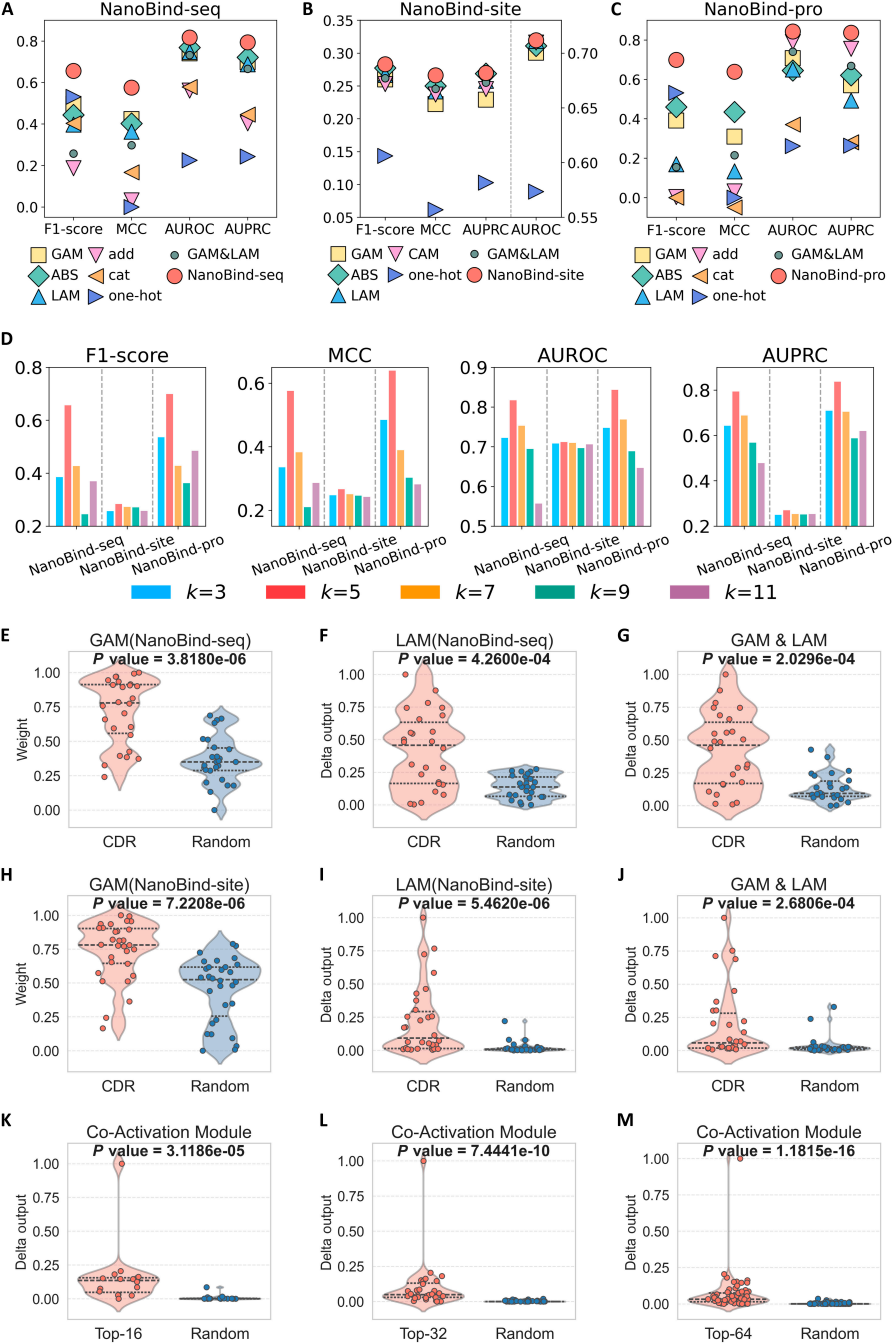
Ablation study and interpretability analysis of NanoBind. (A to C) Ablation studies for NanoBind-seq (A), NanoBind-site (B), and NanoBind-pro (C). GAM, LAM, CAM, and GAM&LAM: ablation of the Global Adaptive Module, Local Adaptive Module, Cross-Assist Module, and both the Global Adaptive Module and Local Adaptive Module, respectively; ABS: absolute position encoding instead of RoPE; one-hot: ESM-2 replaced with one-hot encoding; add/cat: Hadamard product replaced by element-wise addition/concatenation. (D) Contrast experiment of convolution kernel sizes (*k* = 3, 5, 7, 9, 11). (E to G) Interpretability analysis of NanoBind-seq. (E) Attention weights for CDR (red) versus random (blue) residues in Global Adaptive Module. (F) Output score changes after masking CDR (red) versus random (blue) residues in Local Adaptive Module. (G) Output score changes after masking CDR (red) versus random (blue) residues in both Global and Local Adaptive Modules. (H to J) Interpretability analysis of NanoBind-site. (K to M) *U* test results and output score changes after masking the top 16 (K), 32 (L), and 64 (M) dimensions with the highest activation values versus random dimensions in the Co-Activation Module.

First, we replaced the ESM-2 embeddings with one-hot encoding. As shown in Fig. 7A to C, the average F1-score dropped by 30.7%, MCC by 92%, AUROC by 53.6%, and AUPRC by 66.5% (detailed in Tables S13 to S15), indicating that the rich pretrained knowledge effectively compensates for the limited diversity of CDR sequences and antigen types in current datasets, enabling robust generalization that simple one-hot representations completely fail to achieve. Removing the Global Adaptive Module caused a comprehensive decline in all 3 models. Replacing RoPE with absolute position encoding caused a similar decline, demonstrating that relative positional relationships are crucial for modeling long-range dependencies and cooperative patterns among CDRs. Removing the Local Adaptive Module also reduced performance, with F1-score decreases of 39.5% (NanoBind-seq), 5.7% (NanoBind-site), and 75.7% (NanoBind-pro), highlighting its role in capturing local motifs essential for prediction. Removing both the Global Adaptive Module and Local Adaptive Module simultaneously results in a catastrophic performance drop of 60% and 78% in F1-score for NanoBind-seq and NanoBind-pro, respectively, underscoring that the synergistic integration of both global cooperative patterns and local motifs of CDR is essential for capturing the binding profile. We further investigated the effect of different convolution kernel sizes in this module. As shown in Fig. 7D, ablation revealed 5 as optimal for predictive performance (detailed in Tables S16 to S18).

We next ablated the feature fusion strategy between nanobodies and antigens. As illustrated in Fig. 7A and C, replacing the Hadamard product in the Co-Activation Module with concatenation or addition led to a consistent decline in all evaluation metrics for NanoBind-seq and NanoBind-pro (detailed in Tables S13 and S15). This demonstrates that Hadamard multiplication is essential for effective co-activation logic. Finally, the Cross-Assist Module in NanoBind-site improved all 4 metrics by 11.9%, 12.4%, 0.3%, and 10.0%, respectively (Fig. 7B; detailed in Table S14), proving that partner-specific cross-attention is vital for interface residue localization.

### NanoBind captures nanobody–antigen molecular recognition mechanisms

To elucidate the inference decision-making process of NanoBind, we conducted masking experiments [63] and visualization analyses targeting the Global Adaptive Module and Local Adaptive Module in NanoBind-seq and NanoBind-site, as well as the Co-Activation Module in NanoBind-seq and the Cross-Assist Module in NanoBind-site. The results were consistent across all tested samples. Here, we present a detailed analysis using complex 9ETL as a representative case, with the remaining samples provided in Figs. S4 to S8.

In the Global Adaptive Module, a Mann–Whitney *U* test [64] revealed that the attention weights among CDR residues were significantly higher than those among non-CDR residues (Fig. 7E and H; *P* = 3.8 × 10^−6^ and 7.2 × 10^−6^; detailed in Note S9), demonstrating that the module has learned to prioritize long-range CDR cooperativity. In the Local Adaptive Module, CDR residues contribute significantly more to the prediction score than do non-CDR residues (Fig. 7F and I; *P* = 4.3 × 10^−4^ and 5.5 × 10^−6^; detailed in Note S9), showing that the module has learned to focus on local CDR motifs essential for binding. When either CDR or non-CDR residues were simultaneously masked across both modules, the impact of CDR masking on the model's prediction was significantly greater than that of non-CDR masking (Fig. 7G and J; *P* = 2.0 × 10^−4^ and 2.7 × 10^−4^; detailed in Note S9). This demonstrates that the model's decisions are dependent on CDR information, and the insensitivity to non-CDR masking further confirms that the model does not spuriously rely on framework regions. Regarding the fused features derived from the Hadamard product in the Co-Activation Module, masking the top 16, 32, or 64 most activated dimensions significantly affected the binding probability score more than masking an equivalent number of random dimensions (Fig. 7K to M; *P* = 3.1 × 10^−5^, 7.4 × 10^−10^, and 1.2 × 10^−16^; detailed in Note S9), indicating that these dimensions capture the most discriminative interaction features. Furthermore, in the Cross-Assist Module, the attention weights from binding residues of both nanobody and antigen were significantly higher than those from nonbinding residues (Fig. S8A; *P* = 5.4 × 10^−3^; detailed in Note S9), confirming that the module attends to true binding interfaces and captures interaction patterns. Together, these interpretability results empirically validate that NanoBind does not operate as a purely black-box statistical fitter, but autonomously aligns its internal representations with known molecular recognition mechanisms.

## Discussion

This study introduces NanoBind, a unified, mechanism-driven framework that hierarchically profiles nanobody–antigen molecular recognition from binding occurrence to affinity estimation, with information flowing sequentially across 3 tiers. By embedding CDR-dominated binding rules into its architecture, NanoBind bridges the gap between general protein–protein recognition models and nanobody-specific mechanisms. Through Global and Local Adaptive Modules for long- and short-range dependencies, and Cross-Assist and Co-Activation Modules for partner-specific interface localization and cooperative binding, NanoBind achieves robust, interpretable, and data-efficient prediction across binding occurrence, interface residues, and affinity.

A key innovation of this work is the reformulation of affinity prediction from absolute regression to range estimation via pairwise comparison of relative strengths, providing a practical computational route for nanobody potency assessment when quantitative experimental data are scarce. For the nanobody F2–RBD complex, NanoBind-affi prediction aligns with MD simulations and approximates the experimental affinity, validating MD-level precision from sequence alone.

Beyond mechanistic fidelity, NanoBind offers practical advantages in nanobody discovery. Sequence-only inputs and high computational efficiency enable million-scale screening. In virtual screening experiments across 4 antigen systems (GST, lysozyme, pfVAR2CSA, and PD-L1), following NanoBind’s predicted rankings requires a maximum of only a few thousand experimental tests to rapidly identify a true binder among millions of candidates, demonstrating utility as a front-end filter for experimental pipelines. Furthermore, NanoBind provides a refined pool of high-confidence predicted candidates for future experimental prioritization.

Challenges remain for NanoBind. Current NAI datasets are limited in diversity, constraining model generalization to unseen antigen classes and precluding rigorous testing on true or near-binding negative samples. Future work should expand publicly available data and incorporate structural and mutational information. Furthermore, as more experimentally determined *K*_d_ values become available, we aim to refine the reference set with denser anchors, thereby improving the precision of the predicted affinity range and potentially enabling regression-based prediction. Finally, while NanoBind serves as an efficient front-end filter to rapidly exclude low-affinity candidates, it cannot independently pinpoint a clinical nanobody. For comprehensive clinical development, we recommend integrating NanoBind with downstream models to evaluate essential developability profiles alongside affinity (e.g., Seq2Tm [65] for thermal stability and SSH [66] for hydrophobicity).

In summary, NanoBind establishes a mechanism-driven paradigm for nanobody–antigen molecular recognition prediction. By unifying biological insight with deep learning, NanoBind provides a scalable and interpretable foundation for accelerating nanobody discovery and rational design. A user-friendly web server powered by NanoBind is available at http://liulab.top/NanoBind/server (interface details in Figs. S9 and S10).

## Methods

### Data preparation

#### Binding prediction dataset

The training and validation sets (comprising 1,019 and 51 positive samples, respectively) were sourced from the DeepNano study, with positive samples originally derived from the SAbDab-nano [59] database (before 2023 January 24). Negative samples for these sets were generated using the DeepNano cross-mismatching protocol, where mismatches were permitted only for antigens sharing <60% sequence identity. A 1:10 positive-to-negative ratio was maintained, resulting in 11,209 training and 561 validation samples. Separately, an independent test set constructed by Sardar et al. [67] was utilized for benchmarking, which consists of 651 positive samples from sdAb-DB [53] and 1,149 negative samples. These negatives were generated via a stringent mismatching strategy requiring a pairwise antigen edit distance strictly greater than 0.9, as calculated by Clustal Omega [68].

#### Interface residue prediction dataset

The training and validation sets for interface residue prediction were collected from DeepNano. Structural information of the true complexes in the NAI training and validation sets was initially retrieved from the PDB [69]. Residue pairs between the antigen and nanobody within 5 Å were defined as binding residues, yielding 1,019 training and 51 validation samples. We downloaded data from SAbDab-nano from 2023 January 24 to 2025 March 28 and, following the same processing procedure, obtained 439 samples as the test set.

#### Binding affinity prediction dataset

The dataset comprises affinity values (*K*_d_) for 185 nanobody–antigen complexes, collected from the SAbDab-nano database (version dated 2025 March 28) and published literature. Details of the data collection process are provided in Note S4. For the NanoBind-pair (100%), all 185 nanobody–antigen complexes were combined pairwise, and after removing combinations with identical affinity, 16,976 comparison groups were split 6:2:2 into training/validation/test sets. Through data augmentation on the training set by swapping the order of the 2 complexes, we finally obtained 20,370, 3,395, and 3,396 groups, respectively. Due to random splitting at the complex level, complexes in the test set may have appeared in the training or validation sets. For the NanoBind-pair (50%), we employed a 2-stage hold-out strategy. First, 19 of the 185 complexes were held out for validation, and another 19 for test, leaving 147 complexes for training. Subsequently, an additional 18 complexes were sampled from the training pool of 147 and added to each of the held-out sets, resulting in 37 validation and 37 test complexes. This ensures that 50% of the complexes in the test sets are entirely novel to the training and validation sets. Finally, after exhaustive pairwise combination and subsequent filtering of combinations with identical affinity, we obtained 21,708 training, 665 validation, and 663 test sets. For the NanoBind-pair (0%), the 185 complexes were first split 6:2:2. After combination and filtering, this yielded 12,180 training, 666 validation, and 663 test groups. This guarantees that all complexes in the test set are completely unseen during training.

#### Nanobody screening

We collected 59 experimentally validated anti-GST nanobodies from a study [70] and 14 anti-lysozyme, 36 anti-pfVAR2CSA, and 34 anti-PD-L1 nanobodies from the sdAb-DB database. Corresponding antigen sequences were sourced from the UniProt [71] database. One million natural nanobody sequences were randomly sampled as background from the INDI [55] database.

### The architecture of NanoBind

NanoBind is a unified framework that employs a shared NanoBind Encoder and comprises 5 task-specific models: NanoBind-seq and NanoBind-pro for NAI prediction, NanoBind-site for antigen-interface residue identification, NanoBind-pair for relative affinity comparison, and NanoBind-affi for affinity range estimation.

#### NanoBind encoder

Given a nanobody or antigen amino acid sequence S=$\left(s_{1}\;,\;s_{2},\cdots \;,s_{L}\right)$, where *L* is the sequence length and $s_{i}$ denotes the *i*th residue, NanoBind first encodes the sequence using the pretrained protein language model ESM-2. This process generates residue-level embedding:

$$H={\left[h_{1},\;h_{2},\cdots ,\;h_{L}\right]}^{T}\in {\mathbb{R}}^{L\times d_{0}},$$ (1)

where $h_{i}$ represents the embedding vector of residue $s_{i}$, $d_{0}$ is the hidden dimension of the ESM-2 model, and T represents the transposition operation. To prevent overfitting and reduce computational cost, only the last encoder layer of ESM-2 is fine-tuned, while other layers remain frozen.

Considering that nanobody binding is primarily driven by CDRs, 2 parallel pathways are designed to encode CDR-specific information further.

##### Global Adaptive Module

To capture long-range dependencies and relative positional relationships between CDRs, we apply a self-attention enhanced with RoPE. For a residue-level embedding sequence $H\;=\;{\left[h_{1},\;\cdots ,\;h_{L}\right]}^{T}$, NanoBind Encoder first projects it into query, key, and value spaces:

Q=HWQ,K=HWK,V=HWV, (2)

where $W_{Q},W_{K},W_{V}\in {\mathbb{R}}^{d_{0}\times d}$ are learnable parameter matrices and *d* represents the dimension of the hidden layer.

RoPE is introduced to encode relative positional information without breaking sequence permutation equivariance. In RoPE, 2 dimensions are grouped as a 2D pair, and each pair corresponds to a rotation in a 2-dimensional plane. For the *r*th pair $\left(2r:2r+1\right)$, the rotation angle $\theta_{r}$ is defined using sinusoidal positional encoding:

$$\begin{array}{c} \theta_{r}={10,000}^{-2r/d},r=0,1,\cdots ,d/2-1. \end{array}$$ (3)

For a residue embedding $q_{i}=Q_{i,:}\in {\mathbb{R}}^{d}$ at position *i*, the rotated query $\tilde{q_{i}}$ is computed as:

$$\begin{array}{c} \begin{array}{c} {\tilde{q_{i}}}^{\left(2r:2r+1\right)}=\left[\begin{array}{cc} \text{cos}\left(i\theta_{r}\right) & -\text{sin}\left(i\theta_{r}\right)\\ \text{sin}\left(i\theta_{r}\right) & \text{cos}\left(i\theta_{r}\right) \end{array}\right]{q_{i}}^{\left(2r:2r+1\right)},r\\ \;=0,1,\cdots ,d/2-1, \end{array} \end{array}$$ (4)

similarly for $k_{i}=K_{i,:}\in {\mathbb{R}}^{d}$, the rotated key $\tilde{k_{i}}$ is computed as:

$$\begin{array}{c} \begin{array}{c} {\tilde{k_{i}}}^{\left(2r:2r+1\right)}=\left[\begin{array}{cc} \text{cos}\left(i\theta_{r}\right) & -\text{sin}\left(i\theta_{r}\right)\\ \text{sin}\left(i\theta_{r}\right) & \text{cos}\left(i\theta_{r}\right) \end{array}\right]{k_{i}}^{\left(2r:2r+1\right)},r\\ \;=0,1,\cdots ,d/2-1. \end{array} \end{array}$$ (5)

After RoPE, the attention scores are computed as:

$$\begin{array}{c} \alpha_{\mathrm{ij}}=\frac{\text{exp}\left({\tilde{q_{i}}}^{T}\tilde{k_{j}}/\sqrt{d}\right)}{\sum_{r=1}^{L}\text{exp}\left({\tilde{q_{i}}}^{T}\tilde{k_{r}}/\sqrt{d}\right)}, \end{array}$$ (6)

and the context-aware representation of the *i*th residue is obtained by weighted aggregation of the value vectors:

$$\begin{array}{c} h_{i}^{\text{global}}=\sum_{j=1}^{L}\alpha_{\mathrm{ij}}V_{j,:},i=1,2,\cdots ,L. \end{array}$$ (7)

Finally, we obtained the global representation $\begin{array}{c} H^{\text{global}}= \end{array}$$\begin{array}{c} {\left[h_{1}^{\text{global}},\cdots ,\;h_{L}^{\text{global}}\right]}^{T}\in {\mathbb{R}}^{L\times d_{0}} \end{array}$.

##### Local Adaptive Module

Meanwhile, a Local Adaptive Module is employed to capture local functional motifs and structural patterns within the CDRs through 1D convolution.

For the residue-level embeddings $H={\left[h_{1},\;h_{2},\cdots ,\;h_{L}\right]}^{T}$, we applied a 1D-CNN along the sequence dimension. The 1D-CNN utilized multiple filters with a kernel size of k to slide along the sequence, effectively modeling the dependencies between neighboring residues. Let a kernel be represented by $W\in {\mathbb{R}}^{k\times d_{0}}$, where k is the kernel size defining the receptive field. The representation of the *j*th feature for the *i*th residue:

Hi,jlocal=Hi:i+k−1⊗Wj, (8)

where ⊗ represents the convolution operation and this process is carried out in parallel by $d_{0}$ different convolutional kernels $\left\{W_{1},W_{2},\cdots ,W_{d_{0}}\right\}$.

Finally, we obtained the local representation $H^{\text{local}}=$${\left[h_{1}^{\text{local}},\cdots ,\;h_{L}^{\text{local}}\right]}^{T}\in {\mathbb{R}}^{L\times d_{0}}$.

Then, we achieve complementary fusion by concatenating the global representation and local representation to incorporate both global and local information:

$$\begin{array}{c} \mathrm{nef}=[H^{\text{global}};H^{\text{local}}]\in {\mathbb{R}}^{L\times 2d_{0}}, \end{array}$$ (9)

where nef is for residue-level characterization. Meanwhile, we also obtained the adjustment **NEF** at the macromolecular level through average pooling:

$$\begin{array}{c} NEF=\frac{1}{L}\sum_{i=1}^{L}{\mathrm{nef}}_{i,:}\in {\mathbb{R}}^{2d_{0}}. \end{array}$$ (10)

#### NanoBind-seq

For the **NEFs** of nanobody and antigen $NEF_{\mathrm{Nb}},NEF_{\mathrm{Ag}}\in {\mathbb{R}}^{2d_{0}}$ generated by NanoBind Encoder, NanoBind-seq directly models their interactions through Hadamard products, producing an interaction feature that effectively captures the cooperative binding effects between the nanobody and antigen:

$$\begin{array}{c} f=NEF_{\mathrm{Nb}}⨀NEF_{\mathrm{Ag}}\in {\mathbb{R}}^{2d_{0}}, \end{array}$$ (11)

where ⨀ denotes the Hadamard product. The feature is then fed into a multi-layer perceptron (MLP) to predict the binding probability:

$$\begin{array}{c} p_{\mathrm{seq}}=\sigma \left(\mathrm{MLP}\left(f\right)\right), \end{array}$$ (12)

where MLP consists of 5 fully connected layers, with dimensions progressively changing from $2d_{0}$ to 1,024, 512, 256, 128, and finally to 1, each followed by a ReLU activation function, and $\sigma \left(\cdot \right)$ represents the sigmoid function. The output $p_{\mathrm{seq}}$ is a value between 0 and 1, representing the probability of interaction between the nanobody and antigen.

#### NanoBind-site

Given the pre-pooling nanobody embedding ${\mathrm{nef}}_{\mathrm{Nb}}\in {\mathbb{R}}^{m\times 2d_{0}}$, and antigen embedding ${\mathrm{nef}}_{\mathrm{Ag}}\in {\mathbb{R}}^{n\times 2d_{0}}$ (where m and n represent the sequence lengths of the nanobody and antigen, respectively), we employ a Cross-Assist Module that leverages multi-head cross-attention to extract nanobody-guided features and drive the localization of antigen-interface residues. The process of the multi-head cross-attention can be summarized as follows:

$$\begin{array}{c} \mathrm{Ag}=\text{MultiHead}\left({\mathrm{nef}}_{\mathrm{Nb}},{\mathrm{nef}}_{\mathrm{Ag}}\right)=\mathrm{cat}\left({\text{head}}_{1},\cdots ,\;{\text{head}}_{h}\right), \end{array}$$ (13)

where cat represents concatenation and ${\text{head}}_{i}$ is computed as:

$$\begin{array}{c} {\text{head}}_{i}=\text{softmax}\left(\frac{QK^{T}}{\sqrt{d}}\right)V,i=1,2,\cdots ,h, \end{array}$$ (14)

$$\begin{array}{c} \left\{\begin{array}{c} Q={\mathrm{nef}}_{\mathrm{Ag}}W_{Q}\\ K={\mathrm{nef}}_{\mathrm{Nb}}W_{K}\\ V={\mathrm{nef}}_{\mathrm{Nb}}W_{V} \end{array}\right., \end{array}$$ (15)

where $W_{Q},W_{K},W_{V}\in {\mathbb{R}}^{2d_{0}\times d}$ are the learnable parameter matrices for the query, key, and value. h represents the number of heads.

The cross-attention output is fused with the original antigen features through a learnable residual connection:

$$\begin{array}{c} {\mathrm{Ag}}_{\text{out}}=\alpha \cdot \mathrm{Ag}+\left(1-\alpha \right)\cdot {\mathrm{nef}}_{\mathrm{Ag}}, \end{array}$$ (16)

where $\alpha \in \left[0,\;1\right]$ is a learnable parameter controlling the fusion ratio, initialized to 0.5. This design provides a principled mechanism for identifying antigen-interface residues guided by nanobody sequence information.

Subsequently, the global representations of the nanobody and antigen were broadcast and concatenated with the residue-level antigen features:

$$\begin{array}{c} {\mathrm{Ag}}_{\text{fused}}=\mathrm{cat}\left(NEF_{\mathrm{Nb}},\;NEF_{\mathrm{Ag}},\;{\mathrm{Ag}}_{\text{out}},\;{\mathrm{nef}}_{\mathrm{Ag}}\right)\in R^{n\times 8d_{0}}. \end{array}$$ (17)

Finally, the features were deeply extracted through 3 residual units, and after being processed by an MLP and a sigmoid activation function, the probability of each position being a binding residue was obtained:

$$\begin{array}{c} p_{\text{site}}=\sigma \left(\mathrm{MLP}\left({R_{U}}^{\left(3\right)}\left({\mathrm{Ag}}_{\text{fused}}\right)\right)\right)\in {\left[0,1\right]}^{n}, \end{array}$$ (18)

where MLP consists of a fully connected layer, followed by a Dropout layer. ${R_{U}}^{\left(3\right)}$ denote the superposition of 3 layers of Residual Unit, which is defined as:

$$\begin{array}{c} R_{U}\left(X\right)=\text{ReLU}\left(\left(\text{ReLU}\left(XW_{1}+b_{1}\right)\right)W_{2}+b_{2}+X\right), \end{array}$$ (19)

and $W_{1},W_{2},b_{1},b_{2}$ are learnable parameter matrices.

#### NanoBind-pro

For a given antigen sequence, NanoBind-site outputs a binding probability vector $b\in {\left\{0,\;1\right\}}^{n}$ by setting a threshold of 0.5, where n is the length of the antigen sequence, $b_{i}=1$ indicates that the residue at position i is predicted as a binding residue, and $b_{i}=0$ indicates that it is predicted as nonbinding. Subsequently, we established a learnable embedding matrix to represent the semantics of “binding residues” and “nonbinding residues”:

$$\begin{array}{c} e_{i}=E_{b}\left[b_{i}\right]\in {\mathbb{R}}^{d_{p}}, \end{array}$$ (20)

where $E_{b}$ represents the embedding matrix, while $E_{b}\left[0\right]$ denotes the embedding of the nonbinding residue, $E_{b}\left[1\right]$ denotes the embedding of the binding residue, and $d_{p}$ is the embedding dimension. All the residues are embedded and combined to form a matrix:

$$\begin{array}{c} P={\left[e_{1},\cdots ,\;e_{L}\right]}^{T}\in {\mathbb{R}}^{n\times d_{p}}. \end{array}$$ (21)

In order for NanoBind to recognize the sequence order, learnable positional encodings $E_{\mathrm{pos}}\in {\mathbb{R}}^{n\times d_{p}}$ are added for each residue:

$$\begin{array}{c} P^{\prime }=P+E_{\mathrm{pos}}. \end{array}$$ (22)

Next, $P^{\prime }$ is input into the single-layer multi-head Transformer encoder, which contains 8 attention heads, and then obtains the context-enhanced prompt representation through average pooling:

$$\begin{array}{c} p=\frac{1}{n}\sum_{i=1}^{n}\text{MultiHead}{\left(P^{\prime },P^{\prime }\right)}_{i,:}\in {\mathbb{R}}^{d_{p}}. \end{array}$$ (23)

This prompt embedding is concatenated with the original antigen sequence feature $NEF_{\mathrm{Ag}}$ and fused through an MLP layer to obtain the prompt-based antigen representation:

$$\begin{array}{c} NEF_{\mathrm{Ag}}^{\text{prompt}}=\mathrm{MLP}\left(\mathrm{cat}\left(NEF_{\mathrm{Ag}},p\right)\right)\in {\mathbb{R}}^{2d_{0}}, \end{array}$$ (24)

where MLP consists of a fully connected layer, followed by a ReLU activation function.

Finally, the **NEF** of nanobody $NEF_{\mathrm{Nb}}$ is combined with $NEF_{\mathrm{Ag}}^{\text{prompt}}$ through the same Co-Activation Module as NanoBind-seq to produce interaction feature:

$$\begin{array}{c} f=NEF_{\mathrm{Nb}}⨀NEF_{\mathrm{Ag}}^{\text{prompt}}\in {\mathbb{R}}^{2d_{0}}. \end{array}$$ (25)

The feature is then fed into an MLP to predict the interaction probability:

$$\begin{array}{c} p_{\mathrm{pro}}=\sigma \left(\mathrm{MLP}\left(f\right)\right), \end{array}$$ (26)

where MLP consists of 5 fully connected layers, with dimensions progressively changing from $2d_{0}$ to 1,024, 512, 256, 128, and finally to 1, each followed by a ReLU activation function. The output $p_{\mathrm{pro}}$ is a value between 0 and 1, representing the probability of interaction between the nanobody and antigen.

#### NanoBind-pair

For any 2 given nanobody–antigen complexes, we generate their interaction features $\;f_{1},f_{2}\;$ using the same approach as NanoBind-seq, and then employ an MLP to predict which complex exhibits stronger binding affinity:

$$\begin{array}{c} p_{\text{pair}}=\sigma \left(\mathrm{MLP}\left(\mathrm{cat}\left(f_{1},f_{2}\right)\right)\right)\in \left(0,\;1\right). \end{array}$$ (27)

The MLP layer consists of 5 fully connected layers, with dimensions progressively changing from $4d_{0}$ to $2d_{0}$, 1,024, 512, 256, and finally to 1, each followed by a ReLU activation function. A sigmoid function outputs a probability value indicating the likelihood that the *K*_d_ value of the first complex is greater than that of the second complex.

#### NanoBind-affi

NanoBind-affi incorporates an ordered set ${\left\{{\text{Pair}}_{i},\;A_{i}\right\}}_{i=1}^{49}$ as a reference set for affinity range estimation, where ${\text{Pair}}_{i}$ denotes the *i*th nanobody–antigen complex together with its experimentally determined affinity value $A_{i}$, thereby forming 50 contiguous affinity ranges $\left[I_{1}\;,I_{2},\cdots ,I_{50}\right]$, including $\left(0,A_{1}\right)$ and $\left(A_{49},+\infty \right)$. For an input nanobody–antigen complex *T*, its binding affinity is compared with each complex in the reference set sequentially using NanoBind-pair to determine relative strength:

outi=1,NanoBind−pairPairTPairi≥0.50,NanoBind−pairPairTPairi<0.5i=12⋯49. (28)

The score for the affinity of complex *T* belonging to range $I_{j}$ is calculated as follows:

scorej=∑i=149fouti,j=1,2,⋯,50, (29)

where

fouti=outi,i<j1−outi,i≥j. (30)

The range with the highest final score is regarded as the estimated affinity range of the target nanobody–antigen complex:

I^=It, (31)

where

t=argmaxj∈12⋯50scorej. (32)

## Data Availability

All data resources used are freely available from the following databases: Original nanobody–antigen complexes exploited for training were obtained from the SAbDab-nano database at https://opig.stats.ox.ac.uk/webapps/sabdab-sabpred/sabdab/nanobodies/. Part of the nanobody–antigen data for evaluating our NanoBind model was derived from sdAb-DB at https://www.sdab-db.ca/. The large number of natural nanobodies adopted in the case study was downloaded from the INDI2 database at https://naturalantibody.com/indi2/. Sequences of GST, lysozyme, pfVAR2CSA, and PD-L1 were obtained from the UniProt database at https://www.uniprot.org/. The source code files and the processed data for reproducing and evaluating NanoBind are all freely available at the GitHub repository https://github.com/zhaosq17/NanoBind.

## Supplementary Materials

Supplementary 1 Tables S1 to S18 Figs. S1 to S10 Notes S1 to S9
