# Supplementary material for: NanoBind: Mechanism-Driven Deep Learning of Nanobody–Antigen Molecular Recognition
Source: Research (Wash D C). 2026 Jun 23;9:1327. doi: 10.34133/research.1327 (PMC13287458; doi:10.34133/research.1327)
Supplement: Supplementary 1 — Tables S1 to S18 Figs. S1 to S10 Notes S1 to S9 [file research.1327.f1.pdf]

Supplemental Materials

NanoBind: Mechanism-Driven Deep Learning of Nanobody-Antigen Molecular Recognition

Shiqing Zhao[1,†], Yanhao Zhu[1,†], Ruizhou Li[1], Zeyu Xu[1], Mingming Han[2], Jiyun Han[1], Qiuyu Li[1], Mingming Guan[1], Likun Wang[3,4,*], Juntao Liu[1,*], Lijie Xing[2,*]

[1]School of Mathematics and Statistics, Shandong University, Weihai, 264209, China

[2]Shandong Cancer Hospital and Institute, Shandong First Medical University and Shandong Academy of Medical Sciences, Ji'nan, China

[3]Institute of Systems Biomedicine, School of Basic Medical Sciences, Peking University Health Science Center, Beijing 100191, China

[4]Department of Medical Genetics, School of Basic Medical Sciences, Peking University Health Science Center, Beijing 100191, China

[†]These authors contributed equally to this study

[*]To whom correspondence should be addressed

Email addresses:

LW: wanglk@pku.edu.cn

LX: xiaopiao423@126.com

JL: juntaosdu@126.com

Table of contents

Supplementary Tables

Supplementary Figures

Supplementary Notes

Supplementary References

Supplementary Tables

Table S1. Performance (Recall, Precision) of NanoBind-seq, NanoBind-pro, and other compared methods on NAI Prediction.

**Method**	Recall	Precision
DeepNano-seq	0.096 ± 0.084	0.944 ± 0.051
DeepNano	0.143 ± 0.076	0.952 ± 0.022
NABP-LSTM-Att	0.585 ± 0.295	0.528 ± 0.233
D-SCRIPT	0.000 ± 0.001	0.014 ± 0.032
Topsy-Turvy	0.002 ± 0.004	0.041 ± 0.093
PIPR	0 ± 0.00	0 ± 0.00
NanoBind-seq	0.426 ± 0.075	0.860 ± 0.062
NanoBind-pro	0.465 ± 0.048	0.916 ± 0.039

Table S2. Performance (MCC, F1-score, AUROC, and AUPRC) of NanoBind-site and other compared method on Interface Residues Prediction in the Validation Set.

**Method**	MCC	F1-score	AUROC	AUPRC
DeepNano-site	0.705 ± 0.005	0.720 ± 0.004	0.930 ± 0.002	0.774 ± 0.009
NanoBind-site	0.752 ± 0.007	0.767 ± 0.006	0.932 ± 0.010	0.795 ± 0.012

Table S3. Performance of NanoBind-pair (100%) and other compared methods on the Affinity Strength Comparison.

**Method**	MCC	Recall	Precision	F1-score	AUROC	AUPRC	ACC
PIPR	0.020	0.454	0.436	0.445	#	#	0.519
PPA-Pred	-0.105	0.436	0.372	0.402	#	#	0.449
Area-Affinity	-0.041	0.479	0.404	0.438	#	#	0.479
NanoBind-pair (100%)	0.924	0.968	0.945	0.957	0.995	0.994	0.963

100% means that all nanobody-antigen complexes in the test set have appeared in the training set. PIPR, PPA-Pred, and Area-Affinity are regression models, requiring manual collection of their predictions for subsequent conversion into pairwise comparisons; therefore, AUROC and AUPRC values are not available.

Table S4. Performance of NanoBind-pair (50%) and other compared methods on the Affinity

Strength Comparison.

Method	MCC	Recall	Precision	F1-score	AUROC	AUPRC	ACC
PIPR	0.096	0.572	0.567	0.570	#	#	0.549
PPA-Pred	-0.037	0.439	0.502	0.468	#	#	0.480
Area-Affinity	-0.130	0.454	0.448	0.407	#	#	0.433
NanoBind-pair (50%)	0.555	0.809	0.776	0.792	0.851	0.864	0.778

50% overlap indicates that half of the complexes in the test set were included in the training set.

Table S5. Performance of NanoBind-pair (0%) and other compared methods on the Affinity Strength Comparison.

Method	MCC	Recall	Precision	F1-score	AUROC	AUPRC	ACC
PIPR	0.056	0.558	0.402	0.505	#	#	0.522
PPA-Pred	-0.353	0.349	0.228	0.276	#	#	0.312
Area-Affinity	0.009	0.454	0.380	0.414	#	#	0.517
NanoBind-pair (0%)	0.399	0.723	0.583	0.645	0.758	0.615	0.701

0% overlap means that none of the complexes in the test set have appeared in the training set.

Table S6. Affinity range inference reference dataset.

Rank	Complex Name	Kd (M)	Rank	Complex Name	Kd (M)
1	5fv2	7.17E-12	26	1zv5	1.00E-08
2	7JVB	1.00E-11	27	7z1e	1.80E-08
3	5j57	2.00E-11	28	2p43	2.30E-08
4	5hvf	3.00E-11	29	5mzv	3.60E-08
5	5hgg	5.40E-11	30	2vyr	4.40E-08
6	Nb474-TcoALD	6.70E-11	31	1zmy	5.20E-08
7	3p0g	8.40E-11	32	7z1b	6.00E-08
8	1zvy	1.00E-10	33	1g6v	7.20E-08
9	2p4a	1.80E-10	34	7C8W	8.40E-08
10	Nb58-Cry1Ac	3.20E-10	35	4wen	1.00E-07
11	5ENB-VC1	4.10E-10	36	VHH-c3-PCV2	1.80E-07
12	3CNB-VC1	5.50E-10	37	Nb1-CD19	2.90E-07
13	4BNB-VC1	6.20E-10	38	sb45-RBD	3.80E-07
14	5o2u	6.90E-10	39	DA5-BoNTATd	5.56E-07
15	5o05	8.10E-10	40	7KLW	6.90E-07
16	A2-VP40	9.10E-10	41	5imk	8.50E-07
17	4eig	1.00E-09	42	B1-4-OmicronRBD	1.10E-06
18	Nb59-Cry1Ac	1.80E-09	43	M112D-Hoc	2.10E-06
19	aRBD-2	2.60E-09	44	sb68-RBD	5.29E-06

20	1kxq	3.50E-09	45	4weu	5.70E-06
21	7NKT	4.00E-09	46	4wem	6.80E-06
22	CP-B1-MS2	5.00E-09	47	sc180	1.80E-05
23	M24E-CD20-Hoc	5.70E-09	48	M27G-Hoc	0.00044
24	NAB1-N	7.40E-09	49	vhh10-InIA	0.000696
25	7KN6	9.00E-09			

The detailed nanobody and antigen sequences corresponding to each complex can be found in the affinity dataset by querying their names.

Table S7. Predictive performance (Accuracy, Recall, Precision) on 0% test set and homology-based test subsets.

Test Set	Accuracy	Recall	Precision
0% test set	0.667 ± 0.015	0.738 ± 0.069	0.660 ± 0.040
subset1	0.707 ± 0.059	0.709 ± 0.132	0.796 ± 0.147
subset2	0.679 ± 0.054	0.753 ± 0.112	0.704 ± 0.113
subset3	0.646 ± 0.039	0.671 ± 0.129	0.638 ± 0.054
subset4	0.673 ± 0.018	0.771 ± 0.052	0.655 ± 0.043

"0% test set" indicates that the dataset was independently split five times following the 0% splitting strategy in the Methods section. Subsets 1, 2, 3, and 4 denote sets where both antigens share less than 40% sequence identity with the training set, one shares less than 40% while the other shares greater than 40%, one shares less than 40% while the other is 100% identical, and the collection of all remaining samples, respectively.

Table S8. Predictive performance (MCC, F1-score, AUROC, and AUPRC) on 0% test set and homology-based test subsets.

Test Set	F1-score	MCC	AUROC	AUPRC
0% test set	0.694 ± 0.026	0.338 ± 0.032	0.743 ± 0.025	0.738 ± 0.031
subset1	0.736 ± 0.085	0.401 ± 0.116	0.775 ± 0.120	0.838 ± 0.123
subset2	0.720 ± 0.073	0.358 ± 0.106	0.719 ± 0.045	0.758 ± 0.096
subset3	0.648 ± 0.061	0.294 ± 0.068	0.680 ± 0.037	0.717 ± 0.033
subset4	0.706 ± 0.014	0.350 ± 0.051	0.744 ± 0.042	0.749 ± 0.028

Table S9. The intersection of top-scoring GST-targeting candidates predicted by both NanoBind-seq and NanoBind-pro.

INDI_ngs:3595795	INDI_ngs:7591743	INDI_ngs:5170898	INDI_ngs:3644827

INDI_ngs:1311927	INDI_ngs:1176266	INDI_ngs:5476904	INDI_ngs:6707452
INDI_ngs:363198	INDI_ngs:6002846	INDI_ngs:1757787	INDI_ngs:563958
INDI_ngs:6881592	INDI_ngs:6611413	INDI_ngs:1322560	INDI_ngs:10264629
INDI_ngs:8235726	INDI_ngs:1320583	INDI_ngs:345588	INDI_ngs:6814062
INDI_ngs:4696194	INDI_ngs:4251659	INDI_ngs:7456030	INDI_ngs:1767863
INDI_ngs:7431945	INDI_ngs:4694784	INDI_ngs:7595078	INDI_ngs:10876262
INDI_ngs:1259003	INDI_ngs:5761493	INDI_ngs:3343863	INDI_ngs:3498001
INDI_ngs:8141598	INDI_ngs:7463254	INDI_ngs:3521694	INDI_ngs:2481771
INDI_ngs:7735405	INDI_ngs:4127840	INDI_ngs:8836154	INDI_ngs:10373321
INDI_ngs:2581474	INDI_ngs:2450031	INDI_ngs:10570608	INDI_ngs:5006980
INDI_ngs:3535136	INDI_ngs:10925147	INDI_ngs:6056214	INDI_ngs:1736880
INDI_ngs:451465	INDI_ngs:2260624	INDI_ngs:357971	INDI_ngs:1097499
INDI_ngs:11009119	INDI_ngs:6444111	INDI_ngs:9416313	INDI_ngs:90954
INDI_ngs:6608170	INDI_ngs:1905849	INDI_ngs:3953746	INDI_ngs:2136332
INDI_ngs:4244336	INDI_ngs:9799480	INDI_ngs:5148747	INDI_ngs:5119648
INDI_ngs:10449339	INDI_ngs:2435842	INDI_ngs:5517885	INDI_ngs:4671045
INDI_ngs:3889539	INDI_ngs:6933580	INDI_ngs:7359966	INDI_ngs:10635557
INDI_ngs:613575	INDI_ngs:4703741	INDI_ngs:5539079	INDI_ngs:421791
INDI_ngs:4599351	INDI_ngs:10496874	INDI_ngs:7236176	INDI_ngs:7730209
INDI_ngs:948172	INDI_ngs:163454	INDI_ngs:4070404	INDI_ngs:4175909
INDI_ngs:4568275	INDI_ngs:1881751	INDI_ngs:1133446	
INDI_ngs:10791556	INDI_ngs:204974	INDI_ngs:1042057	

IDs starting with “INDI_ngs:” denote nanobody sequences sourced from the INDI (Integrated Database of Nanobodies for Immunoinformatics) database. The numeric suffix (e.g., 8518456) is the unique identifier from the original NGS data source. These sequences can be retrieved by searching the respective ID on the INDI database or its source repository.

Table S10. The intersection of top-scoring lysozyme-targeting candidates predicted by both NanoBind-seq and NanoBind-pro.

INDI_ngs:10060584

Table S11. The intersection of top-scoring pfVAR2CSA-targeting candidates predicted by both NanoBind-seq and NanoBind-pro.

INDI_ngs:4827052	INDI_ngs:9307621	INDI_ngs:2141277	INDI_ngs:9962958
INDI_ngs:1240064	INDI_ngs:9524283	INDI_ngs:2820200	INDI_ngs:6151008
INDI_ngs:3286678	INDI_ngs:4115776	INDI_ngs:8995561	INDI_ngs:469506
INDI_ngs:10122393	INDI_ngs:2380258	INDI_ngs:3882547	INDI_ngs:5638650
INDI_ngs:1779561	INDI_ngs:2044913	INDI_ngs:155665	INDI_ngs:5500583
INDI_ngs:5059843	INDI_ngs:1991869	INDI_ngs:6503381	INDI_ngs:7493770

INDI_ngs:2918835	INDI_ngs:3224032	INDI_ngs:7968403	INDI_ngs:7237543
INDI_ngs:9965441	INDI_ngs:9947242	INDI_ngs:7243981	INDI_ngs:3823286
INDI_ngs:5264970	INDI_ngs:1066939	INDI_ngs:2821097	INDI_ngs:7853564
INDI_ngs:9687088	INDI_ngs:7143025	INDI_ngs:5399782	INDI_ngs:561489
INDI_ngs:3750898	INDI_ngs:1590872	INDI_ngs:7226484	
INDI_ngs:3655478	INDI_ngs:5035187	INDI_ngs:5515135	

Table S12. The intersection of top-scoring PD-L1-targeting candidates predicted by both NanoBind-seq and NanoBind-pro.

INDI_ngs:7831226	INDI_ngs:10654747	INDI_ngs:6006669	INDI_ngs:5862022
INDI_ngs:6602902	INDI_ngs:839126	INDI_ngs:1589054	INDI_ngs:6793500
INDI_ngs:3277529	INDI_ngs:4833090	INDI_ngs:7318758	INDI_ngs:880437
INDI_ngs:5828125	INDI_ngs:2859681	INDI_ngs:3788223	INDI_ngs:9878201
INDI_ngs:1713211	INDI_ngs:6485256	INDI_ngs:1362351	INDI_ngs:6277195
INDI_ngs:893272	INDI_ngs:6054773	INDI_ngs:4619092	INDI_ngs:7535935
INDI_ngs:1493086	INDI_ngs:9782037	INDI_ngs:4028958	INDI_ngs:4927279
INDI_ngs:2039981	INDI_ngs:3142724	INDI_ngs:3344207	INDI_ngs:295496
INDI_ngs:4294264	INDI_ngs:4614989	INDI_ngs:9089655	INDI_ngs:3811672
INDI_ngs:4691041	INDI_ngs:4995996	INDI_ngs:7311969	INDI_ngs:3573110
INDI_ngs:5304624	INDI_ngs:6792407	INDI_ngs:4513342	INDI_ngs:8265160
INDI_ngs:6292971	INDI_ngs:4787484	INDI_ngs:2382628	INDI_ngs:484288
INDI_ngs:637423	INDI_ngs:6467313	INDI_ngs:1715675	INDI_ngs:1531426
INDI_ngs:4927625	INDI_ngs:4367593	INDI_ngs:7760920	INDI_ngs:7941823
INDI_ngs:5955065	INDI_ngs:6593227	INDI_ngs:9552145	INDI_ngs:7287625
INDI_ngs:5535466	INDI_ngs:5229967	INDI_ngs:6316895	INDI_ngs:1674260
INDI_ngs:2322978	INDI_ngs:1947820	INDI_ngs:3227770	INDI_ngs:10592893
INDI_ngs:6281997	INDI_ngs:1234070	INDI_ngs:9035958	
INDI_ngs:2160744	INDI_ngs:2087018	INDI_ngs:9858975	
INDI_ngs:2141529	INDI_ngs:367329	INDI_ngs:932124	

Table S13. Performance metrics of ablation studies with different settings on NanoBind-seq.

**Ablation**	MCC	Recall	Precision	F1-score	AUROC	AUPRC
original	0.576	0.515	0.905	0.656	0.817	0.793
GAM	0.424	0.352	0.845	0.497	0.740	0.698
ABS	0.403	0.297	0.881	0.444	0.769	0.722
LAM	0.363	0.258	0.862	0.397	0.747	0.688
GAM&LAM	0.2974	0.149	0.9417	0.2573	0.7324	0.6654
add	0.032	0.121	0.405	0.187	0.561	0.403

cat	0.167	0.336	0.503	0.403	0.578	0.445
one-hot	0.000	1.000	0.362	0.531	0.225	0.244

Original indicates the complete NanoBind-seq model; GAM, LAM, CAM, and GAM&LAM represent the ablation of the Global Adaptive Module, Local Adaptive Module, Cross-Assist Module, and both the Global Adaptive Module and Local Adaptive Module, respectively; ABS indicates using absolute position encoding instead of RoPE; one-hot signifies the replacement of ESM-2 with one-hot encoding; add and cat denote the substitution of the Hadamard product with element-wise addition and concatenation.

Table S14. Performance metrics of ablation studies with different settings on NanoBind-site.

**Ablation**	MCC	Recall	Precision	F1-score	AUROC	AUPRC
original	0.266	0.209	0.441	0.283	0.712	0.270
CAM	0.237	0.183	0.411	0.253	0.710	0.245
GAM	0.222	0.210	0.340	0.260	0.700	0.229
ABS	0.250	0.214	0.395	0.277	0.707	0.269
LAM	0.242	0.202	0.392	0.267	0.711	0.258
GAM&LAM	0.2454	0.1901	0.4195	0.2617	0.7028	0.2551
one-hot	0.062	0.176	0.121	0.144	0.574	0.103

Original indicates the complete NanoBind-site model.

Table S15. Performance of ablation studies with different settings on NanoBind-pro.

**Ablation**	MCC	Recall	Precision	F1-score	AUROC	AUPRC
original	0.639	0.547	0.967	0.699	0.843	0.836
GAM	0.309	0.264	0.751	0.391	0.708	0.570
ABS	0.434	0.306	0.930	0.460	0.645	0.620
LAM	0.133	0.098	0.621	0.170	0.650	0.493
GAM&LAM	0.2145	0.0845	0.9167	0.1547	0.7408	0.6679
add	0.031	0.002	1.000	0.003	0.790	0.755
cat	-0.050	0.000	0.000	0.000	0.371	0.281
one-hot	0.000	1.000	0.362	0.531	0.261	0.263

Original indicates the complete NanoBind-pro model.

Table S16. Performance of ablation studies with different convolution kernel sizes on NanoBind-seq.

**Ablation**	MCC	Recall	Precision	F1-score	AUROC	AUPRC
k=5 (original)	0.576	0.515	0.905	0.656	0.817	0.793
k=3	0.335	0.252	0.816	0.385	0.723	0.643
k=7	0.382	0.284	0.861	0.427	0.753	0.687
k=9	0.209	0.148	0.716	0.245	0.694	0.568
k=11	0.286	0.247	0.729	0.369	0.557	0.477

k refers to the size of the convolutional kernel.

Table S17. Performance of ablation studies with different convolution kernel sizes on NanoBind-site.

**Ablation**	MCC	Recall	Precision	F1-score	AUROC	AUPRC
k=5 (original)	0.266	0.209	0.441	0.283	0.712	0.270
k=3	0.247	0.182	0.439	0.257	0.708	0.249
k=7	0.250	0.204	0.409	0.273	0.710	0.253
k=9	0.246	0.206	0.396	0.271	0.696	0.251
k=11	0.242	0.187	0.416	0.258	0.706	0.253

k refers to the size of the convolutional kernel.

Table S18. Performance of ablation studies with different convolution kernel sizes on NanoBind-pro.

**Ablation**	MCC	Recall	Precision	F1-score	AUROC	AUPRC
k=5 (original)	0.639	0.547	0.967	0.699	0.843	0.836
k=3	0.484	0.378	0.918	0.535	0.747	0.708
k=7	0.389	0.283	0.876	0.427	0.769	0.704
k=9	0.303	0.237	0.774	0.362	0.689	0.587
k=11	0.281	0.407	0.598	0.485	0.646	0.620

k refers to the size of the convolutional kernel.

Supplementary Figures

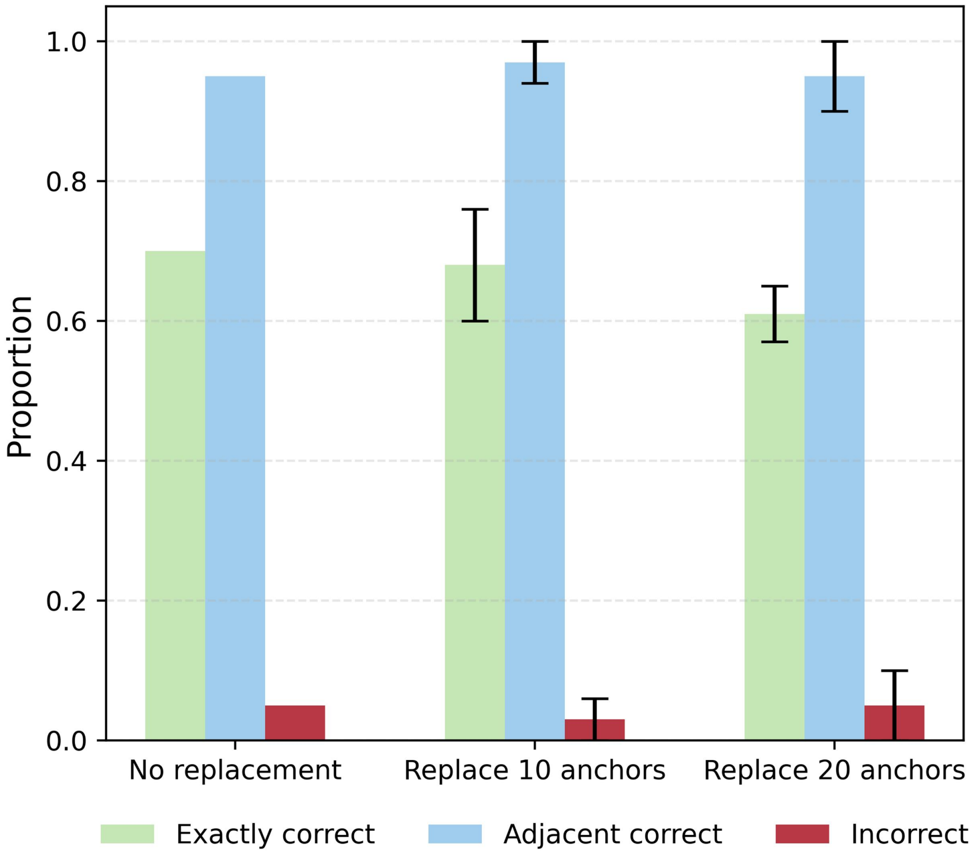

Figure S1. Performance of Affinity Range Estimation Under Anchor Replacement. The horizontal axis represents three conditions: no anchor replacement, random replacement of 10 anchors (5 replicates), and random replacement of 20 anchors (5 replicates). The vertical axis shows the proportion of predictions (0-1). Green bars ("Exactly correct") indicate predictions where the estimated interval exactly matches the true interval. Blue bars ("Adjacent correct") indicate predictions where the estimated interval falls within the adjacent interval to the true range (error $\leq$ 1 interval). Red bars ("Incorrect") indicate predictions where the estimated interval deviates by two or more intervals from the true range. Error bars represent the standard deviation across replicate experiments (the no-replacement condition had a single run and therefore no error bars). The results demonstrate that random anchor replacement has only a minor effect on affinity range estimation, with the vast majority of predictions ($\geq$95%) falling within the true interval or its immediate neighbor, indicating overall stability of the interval estimates.

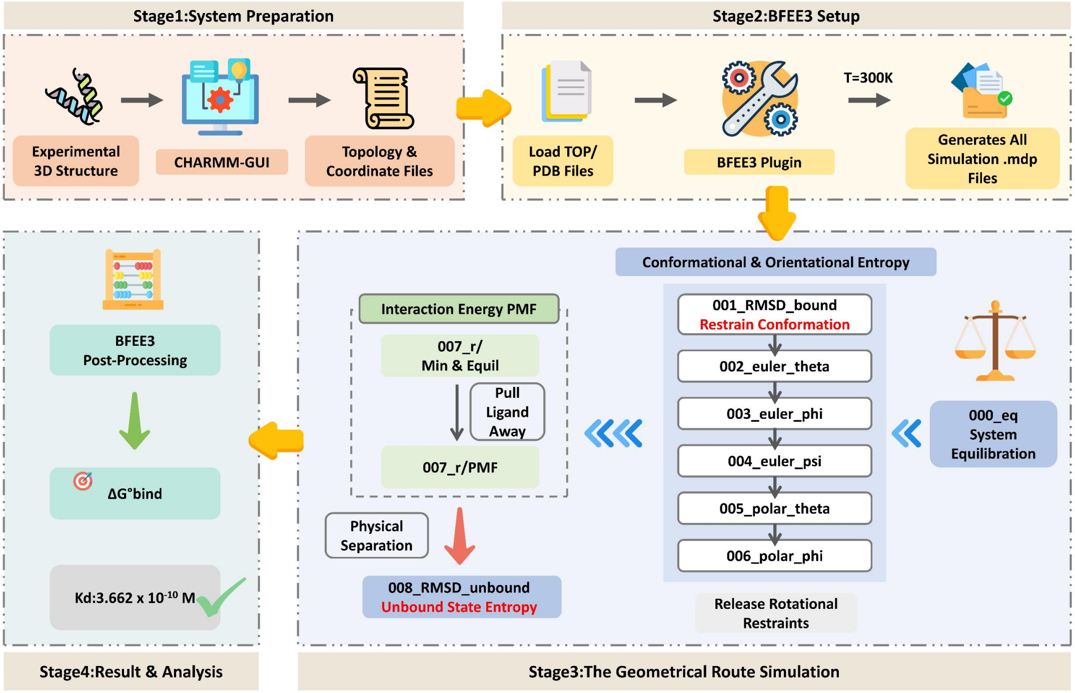

Figure S2. Detailed Flowchart of Molecular Dynamics Simulation. First, a simulation system was constructed using CHARMM-GUI, and the BFEE3 protocol was employed for constrained sampling; subsequently, the spatial constraints on the ligand were progressively released to sample its different degrees of freedom.

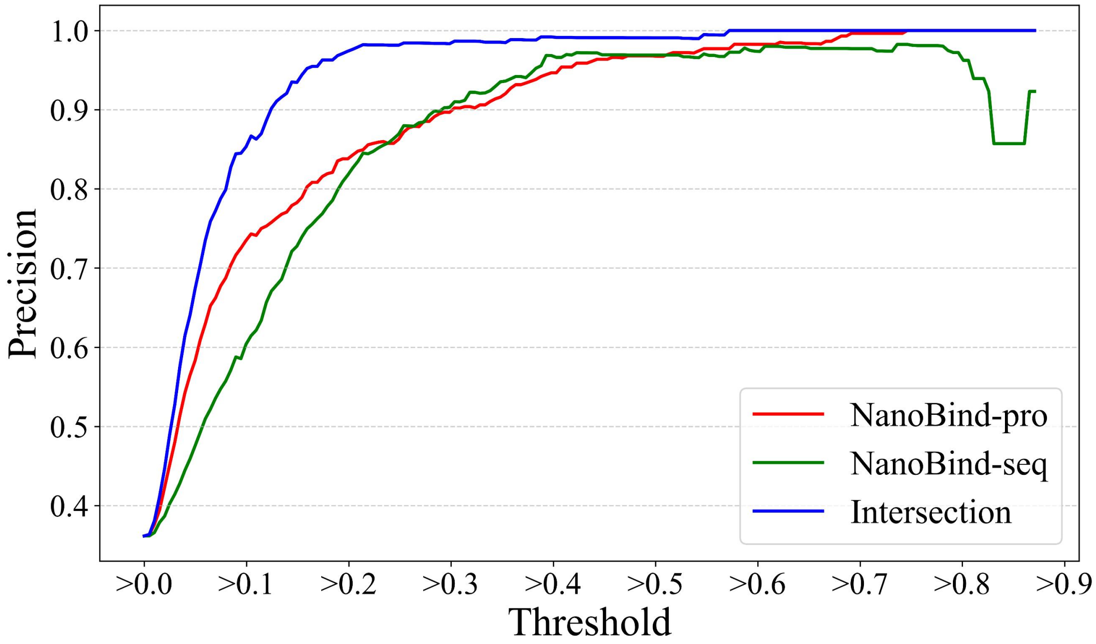

Figure S3. Precision Dynamics of NanoBind-seq and NanoBind-pro Across Varying Prediction Thresholds. The horizontal axis represents the threshold value for predicting positive

samples, and the vertical axis indicates the precision. The red and green curves, respectively, illustrate the precision changes of the NanoBind-pro and NanoBind-seq models at different thresholds. The blue curve demonstrates the precision calculated at various thresholds when both models are applied simultaneously, with their intersection serving as the predicted positive samples.

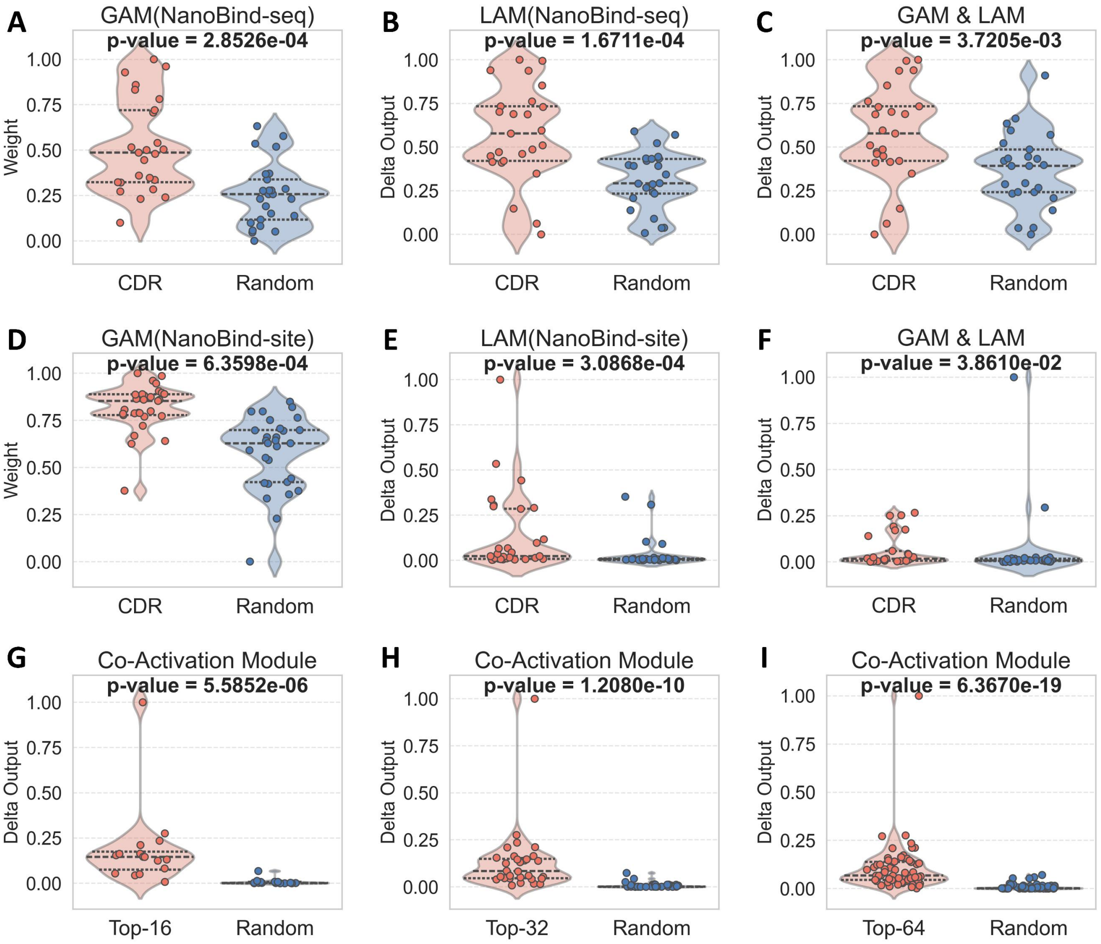

Figure S4. Interpretability Analysis Results of Complex 8XK2. The nanobody chain (h) with three CDR regions of lengths 7, 6, and 12 residues, harboring 29 binding residues. The corresponding antigen chain (g) contains 29 binding residues. (A-C) Interpretability analysis of NanoBind-seq. (A) Attention weights for CDR (red) versus random (blue) residues in Global Adaptive Module. (B) Output score changes after masking CDR (red) versus random (blue) residues in Local Adaptive Module. (C) Output score changes after masking CDR (red) versus random (blue) residues in both Global and Local Adaptive Modules. (D-F) Interpretability

analysis of NanoBind-site. (G-I) U-test results, and scatter distributions of output score changes after masking the top 16 (G), 32 (H), and 64 (I) dimensions with the highest activation values versus random dimensions in the Co-Activation Module. A Mann-Whitney U test showed a significantly higher attention weight among CDRs than non-CDR residues ($p = 2.9 \times 10^{-4}$, 6.4×10^{-4}), confirming that the Global Adaptive Module effectively focuses on the nanobody CDR regions. In the Local Adaptive Module, masking CDR residues caused a significantly greater impact on the output ($p = 1.7 \times 10^{-4}$, 3.1×10^{-4}), verifying the module's specific responsiveness to CDR regions. Together, when masking both CDR and non-CDR residues simultaneously in these two modules, the impact of CDR residues on the model's prediction was significantly greater than that of non-CDR residues ($p = 3.7 \times 10^{-3}$, 3.9×10^{-2}). This demonstrates that the model's decisions are causally dependent on CDR information, and the insensitivity to non-CDR masking further confirms that the model does not spuriously rely on framework regions. In the Co-Activation Module, masking the top 16, 32, and 64 activated dimensions caused significantly greater impact on the output score ($p=5.6\times10^{-6}$, 1.2×10^{-10}, 6.4×10^{-19}, respectively), confirming these dimensions capture the most discriminative interaction features.

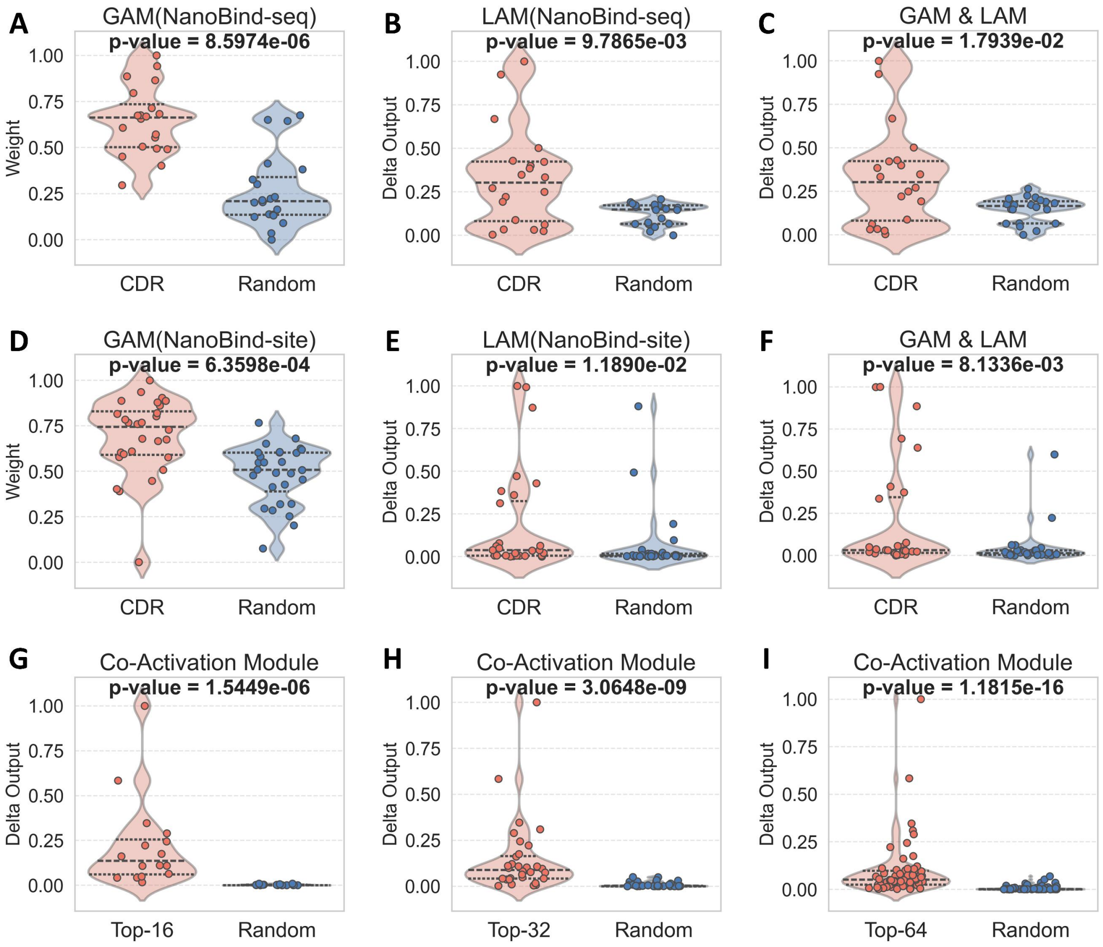

Figure S5. Interpretability Analysis Results of Complex 8OUD. The nanobody chain (d) with three CDR regions of lengths 7, 5, and 8 residues, harboring 28 binding residues. The corresponding antigen chain (a) contains 33 binding residues. (A-C) Interpretability analysis of NanoBind-seq. (A) Attention weights for CDR (red) versus random (blue) residues in Global Adaptive Module. (B) Output score changes after masking CDR (red) versus random (blue) residues in Local Adaptive Module. (C) Output score changes after masking CDR (red) versus random (blue) residues in both Global and Local Adaptive Modules. (D-F) Interpretability analysis of NanoBind-site. (G-I**)** U-test results, and scatter distributions of output score changes after masking the top 16 (G), 32 (H), and 64 (I) dimensions with the highest activation values versus random dimensions in the Co-Activation Module.

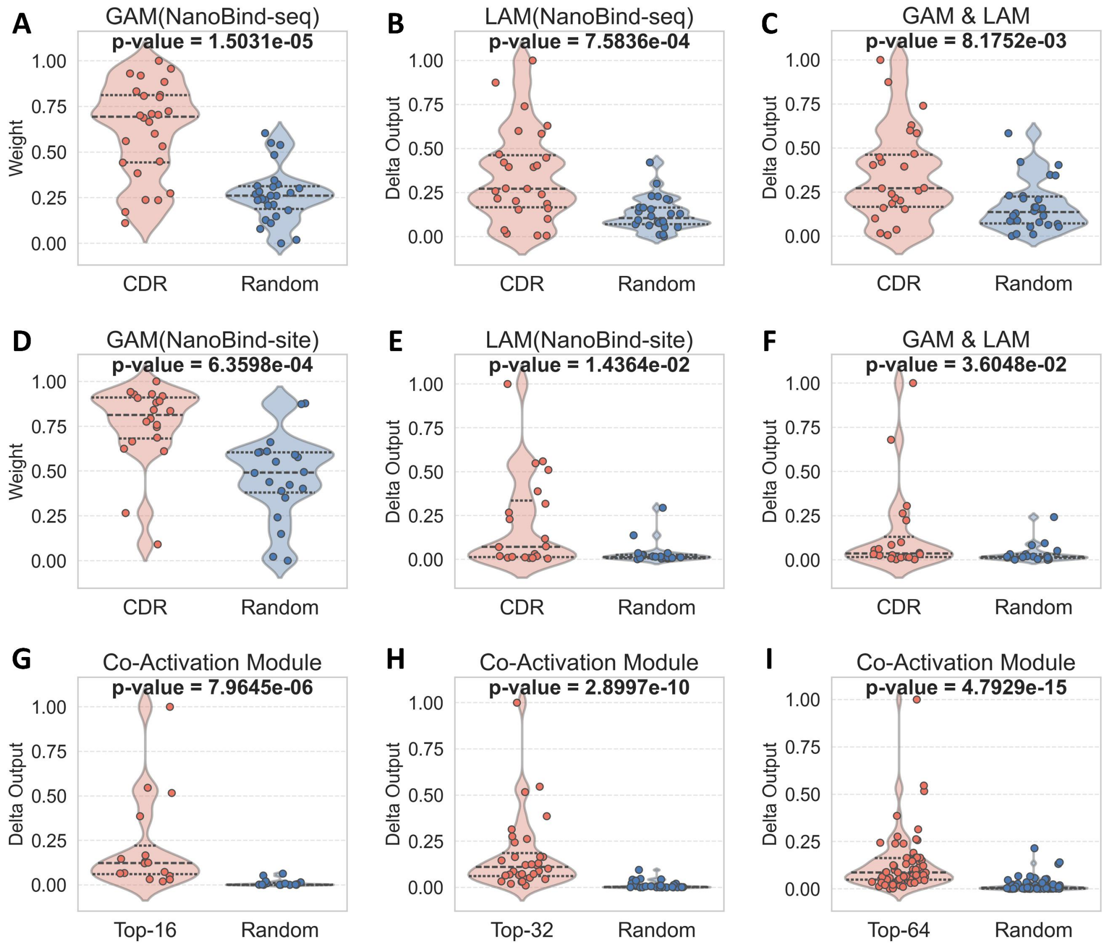

Figure S6. Interpretability Analysis Results of Complex 7ZOX. The nanobody chain (c) with three CDR regions of lengths 7, 6, and 14 residues, harboring 20 binding residues. The corresponding antigen chain (a) contains 21 binding residues. (A-C) Interpretability analysis of NanoBind-seq. (A) Attention weights for CDR (red) versus random (blue) residues in Global Adaptive Module. (B) Output score changes after masking CDR (red) versus random (blue) residues in Local Adaptive Module. (C) Output score changes after masking CDR (red) versus random (blue) residues in both Global and Local Adaptive Modules. (D-F) Interpretability analysis of NanoBind-site. (G-I**)** U-test results, and scatter distributions of output score changes after masking the top 16 (G), 32 (H), and 64 (I) dimensions with the highest activation values versus random dimensions in the Co-Activation Module.

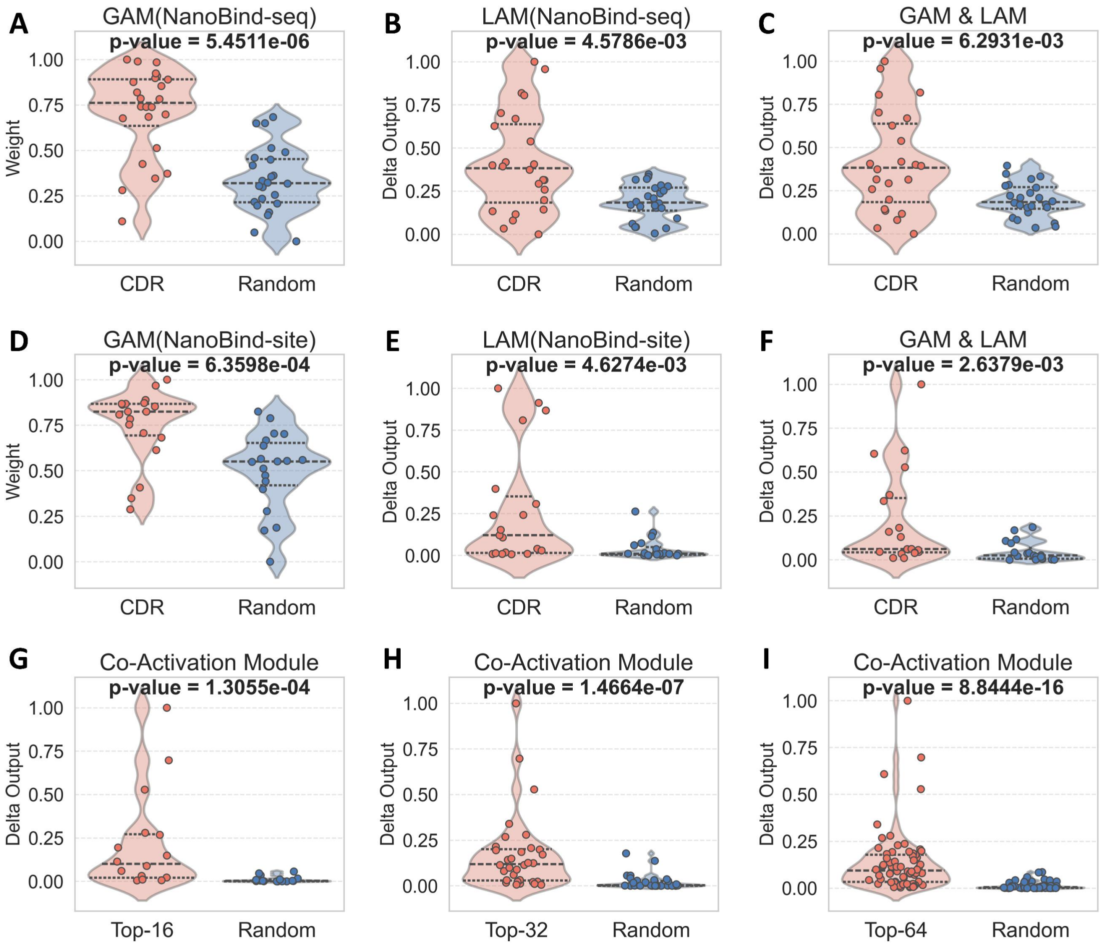

Figure S7. Interpretability Analysis Results of Complex 8EE2. The nanobody chain (h) with three CDR regions of lengths 7, 5, and 12 residues, harboring 19 binding residues. The corresponding antigen chain (f) contains 35 binding residues. (A-C) Interpretability analysis of NanoBind-seq. (A) Attention weights for CDR (red) versus random (blue) residues in Global Adaptive Module. (B) Output score changes after masking CDR (red) versus random (blue) residues in Local Adaptive Module. (C) Output score changes after masking CDR (red) versus random (blue) residues in both Global and Local Adaptive Modules. (D-F) Interpretability analysis of NanoBind-site. (G-I**)** U-test results, and scatter distributions of output score changes after masking the top 16 (G), 32 (H), and 64 (I) dimensions with the highest activation values versus random dimensions in the Co-Activation Module.

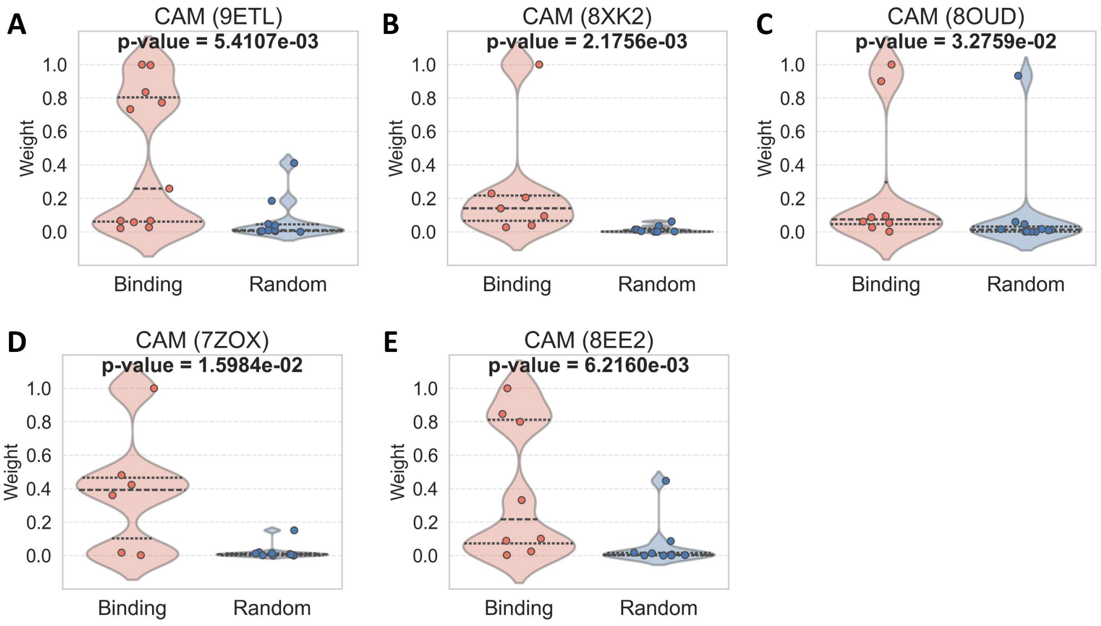

Figure S8. Interpretability Analysis Results of Cross-Assist Module. (A-E) Attention weights for binding (red) versus random (blue) residues of complex 9ETL (A), 8XK2 (B), 8OUD (C), 7ZOX (D), 8EE2 (E). The weight distribution for binding-residue groups was significantly higher than that for non-binding groups in the Cross-Assist Module of NanoBind-site. This result proves that the Cross-Assist Module selectively leverages binding-competent nanobody residues to precisely localize antigen-interface residues.

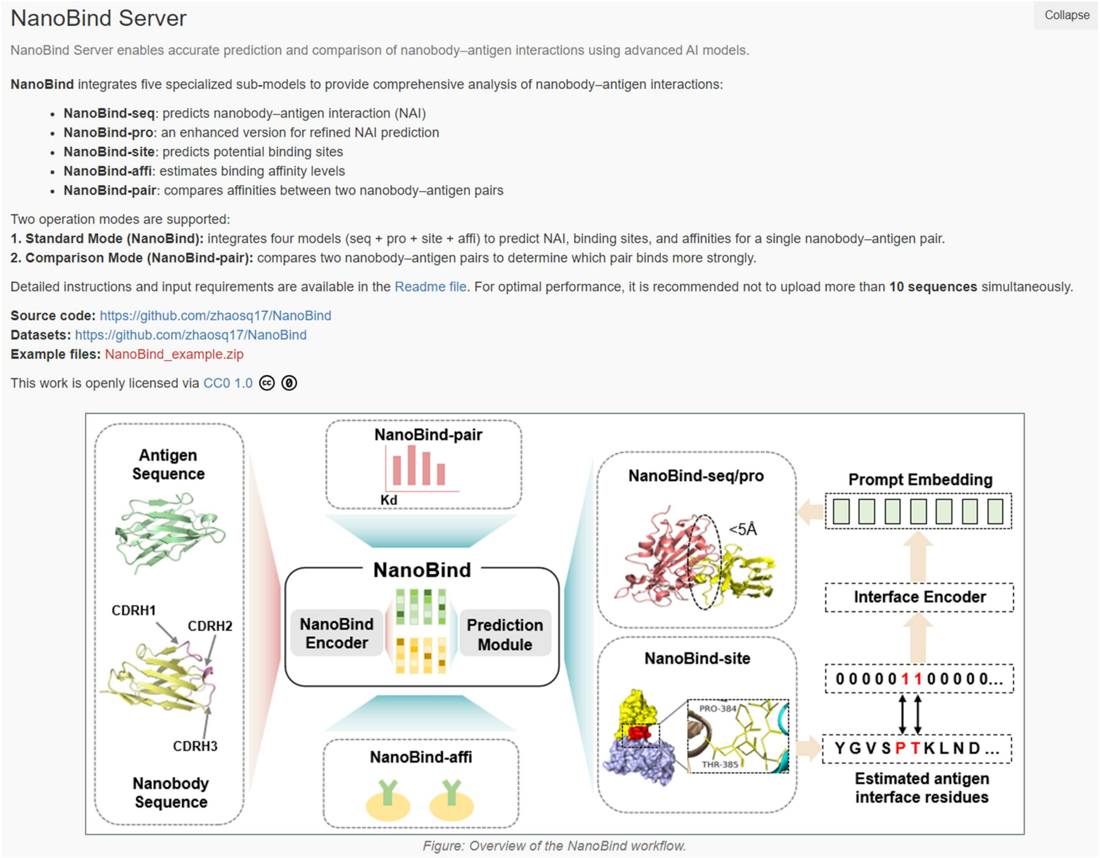

NanoBind Server

Collapse

NanoBind Server enables accurate prediction and comparison of nanobody–antigen interactions using advanced AI models.

NanoBind integrates five specialized sub-models to provide comprehensive analysis of nanobody–antigen interactions:

- **NanoBind-seq**: predicts nanobody–antigen interaction (NAI)
- **NanoBind-pro**: an enhanced version for refined NAI prediction
- **NanoBind-site**: predicts potential binding sites
- **NanoBind-affi**: estimates binding affinity levels
- **NanoBind-pair**: compares affinities between two nanobody–antigen pairs

Two operation modes are supported:
1. Standard Mode (NanoBind): integrates four models (seq + pro + site + affi) to predict NAI, binding sites, and affinities for a single nanobody–antigen pair.
2. Comparison Mode (NanoBind-pair): compares two nanobody–antigen pairs to determine which pair binds more strongly.

Detailed instructions and input requirements are available in the Readme file. For optimal performance, it is recommended not to upload more than **10 sequences** simultaneously.

Source code: https://github.com/zhaosq17/NanoBind
Datasets: https://github.com/zhaosq17/NanoBind
Example files: NanoBind_example.zip

This work is openly licensed via CC0 1.0

Figure: Overview of the NanoBind workflow.

Figure S9. Web Server of NanoBind. The webpage of NanoBind's online server.

Select NanoBind Mode

(●) **Standard Mode:** NanoBind (Seq + Pro + Site + Affi)
( ) **Comparison Mode:** NanoBind-pair (Compare two pairs)

Upload FASTA Files

Please upload your input files in FASTA format (.fasta or .fa).

Nanobody:

```
>normal-test-nanobody
QVQLQESGGGSVQAGGSLRLSCVASGLT
FSIYRMYWYRQAPGKACELVSLIIPDGT
TTYADSVKGRFTISRDDAKNTVYLQMNS
LEPEDTAVYYCAASTAGNWPRACTDFVY
QGQGTQVTVSS
>normal-test2-nanobody
EVQLLVSGGGLVQPGGSLRLSCAASGFT
FKAYPMMWVRQAPGKGLEWVSEISPSGS
YTYYADSVKGRFTISRDNSKNTLYLQMN
```

NanoBind_test_nanobody.fasta
(597.00 B)

NanoBind_test_nanobody.fasta | Remove | Upload | Browse …

Antigen:

```
>normal-test-antigen
MSKGEELFTGVVPILVELDGDVNGHKFS
VSGEGEGDATYGKLTLKFICTTGKLPVP
WPTLVTTFSYGVQCFSRYPDHMKQHDFF
KSAMPEGYVQERTIFFKDDGNYKTRAEV
KFEGDTLVNRIELKGIDFKEDGNILGHK
LEYNYNSHNVYIMADKQKNGIKVNFKIR
HNIEDGSVQLADHYQQNTPIGDGPVLLP
DNHYLSTQSALSKDPNEKRDHMVLLEFV
TAAGITHGMDELYK
```

NanoBind_test_antigen.fasta
(1.11 KB)

NanoBind_test_antigen.fasta | Remove | Upload | Browse …

Submit

NanoBind Prediction Results

Download Full CSV

Binding Predictions (Only Show Top 5 Pairs)

Nanobody	Antigen	NanoBind_Seq_score	NanoBind_Seq_class	NanoBind_Pro_score	NanoBind_Pro_class	All Predicted Affinity interval
normal-test-nanobody	normal-test-antigen	0.447252	1	0.777104	1	[3.20e-09, 3.50e-09] M
normal-test2-nanobody	norml-test2-antigen	0.334553	1	0.875707	1	< 7.17e-12 M
negative-test-nanobody	negative-test-antigen	0.087162	0	0.00851	0	NA
multi-interval-test-nanobody	multi-interval-test-antigen	0.41596	1	0.890454	1	[1.01e-06, 5.70e-06] M; > 6.80e-06 M

Binding Sites Predictions (Only Show Top 5 Pairs)

Antigen	Site_positions & Amino acid type
normal-test-antigen	151:Y; 166:K; 168:R; 176:V; 207:L
norml-test2-antigen	7:M; 10:Y; 11:Q; 12:R; 14:Y; 15:C; 16:H; 51:N; 52:D; 55:L; 92:E; 93:C; 94:R; 95:P
negative-test-antigen	NA
multi-interval-test-antigen	53:T; 54:D; 55:Y; 56:E; 57:G; 81:K; 83:A; 84:E; 85:G; 86:T; 87:K; 223:P; 225:N; 236:S; 238:D

Figure S10. Usage and Result Demonstration of the NanoBind Online Service. The NanoBind web server provides an interactive interface for submitting input data and viewing results. The result page displays a table containing the predicted binding probability, antigen-interface residues, and the predicted affinity range (or relative affinity strength in the NanoBind-pair mode). In addition, users can download the result file directly from the top of the table.

Supplementary Notes

Supplementary Note 1 | Parameter settings of other compared methods

All computational workflows were executed on a server equipped with dual Intel® Xeon® Silver 4210R CPUs and an NVIDIA RTX A6000 GPU to ensure reproducible performance metrics.

(1) DeepNano-seq, DeepNano, and DeepNano-site. The training and evaluation procedures for the three models were meticulously executed following their original configurations. All hyperparameters and architecture components were kept unchanged to preserve methodological integrity. The training data were sourced exclusively from the authors' provided dataset (https://github.com/ddd9898/DeepNano/tree/main/data). We rigorously trained the models for 10 epochs (DeepNano-seq and DeepNano) and 200 epochs (DeepNano-site), selecting the model with the highest validation performance for final testing and comparison.

(2) NABP-LSTM-Att. The training and evaluation procedures for the model were meticulously executed following their original configurations. All hyperparameters were strictly maintained at their recommended default values to ensure consistency with the original implementations. We retrained it for 100 epochs using our NAI data and selected the final model for testing and comparison. For each nanobody, we took the average of the predicted scores for its three CDR-antigen pairs as the final NAI probability.

(3) D-SCRIPT and Topsy-Turvy. The training and evaluation procedures for both D-SCRIPT and Topsy-Turvy were meticulously carried out following their official documentation (https://d-script.readthedocs.io/en/stable/usage.html). All hyperparameters were strictly maintained at their recommended default values to ensure consistency with the original implementations. We retrained both models for 10 epochs using our NAI data and selected the model with the highest validation performance for final testing and comparison.

(4) PIPR. For PIPR implementation, we utilized the authors' codebase (https://github.com/muhaochen/seq_ppi.git). All hyperparameters were strictly maintained at their recommended default values to ensure consistency with the original implementations. We retrained the model for 10 epochs on our datasets for NAI prediction and affinity prediction tasks, respectively, and selected the model with the highest validation performance

for final testing and comparison.

(5) PPA-Pred, Area-Affinity. PPA-Pred and Area-Affinity were both accessed through their online web servers: PPA-Pred (https://www.iitm.ac.in/bioinfo/PPA_Pred/prediction.html#) and Area-Affinity (https://affinity.cuhk.edu.cn/). For PPA-Pred, the "Antigen-Antibody" option was selected, and the amino acid sequences of the nanobody-antigen pairs were manually submitted to obtain the predicted binding affinities. For Area-Affinity, the "Antibody-Protein Antigen" option was used, which requires structural information as input. Complex structures were retrieved from the PDB database, and for cases lacking available PDB entries, structures were generated using Chai-1. Since both servers only output individual affinity predictions, for each input nanobody-antigen pair (A and B), we manually submitted the data to both servers and compared their predicted binding affinities to determine the relative affinity ranking between the two complexes.

Supplementary Note 2 | Descriptions of performance metrics

In this study, we used the following evaluation metrics to measure the predicted performance: Precision, Recall, F1-score, Matthews correlation coefficient (MCC), area under the receiver operating characteristic curve (AUROC), area under the precision-recall curve (AUPRC), and accuracy (ACC). Denoting true positives (TP), true negatives (TN), false positives (FP), and false negatives (FN):

$$Precision = \frac{TP}{TP + FP},$$

$$Recall = \frac{TP}{TP + FN},$$

$$F1 - score = 2 \times \frac{Precision \times Recall}{Precision + Recall},$$

$$MCC = \frac{TP \times TN - FP \times FN}{\sqrt{(TP + FP) \times (TP + FN) \times (TN + FP) \times (TN + FN)}},$$

$$Accuracy = \frac{TP + TN}{TP + TN + FP + FN}.$$

The AUROC is calculated as the area under the ROC curve. A ROC curve shows the trade-off between true positive rate (TPR) and false positive rate (FPR) across different decision thresholds. The AUPRC is calculated as the area under the PR curve. A PR curve is a graph with Precision values on the y-axis and Recall values on the x-axis across different

decision thresholds.

Supplementary Note 3 | Decision threshold optimization of NanoBind

For all three tasks, the decision threshold was determined based on the optimal F1-score. Specifically, threshold values ranging from 0.0 to 1.0 (with a step size of 0.1) were systematically evaluated on the validation set, and the threshold achieving the highest F1-score was selected for final metric computation. All threshold-dependent classification metrics, including precision, recall, accuracy, MCC, and the F1-score itself, were computed using predictions generated at this optimized threshold.

Supplementary Note 4 | Affinity data collection and partitioning strategy

We collected a total of 185 experimentally annotated nanobody-antigen complexes with Kd values from two sources. First, 71 complexes were sourced from the SAbDab-nano database (version dated March 28, 2025). Next, we performed a PubMed search using the combined keywords “nanobody” AND “SPR”, which returned 66 relevant articles. Most of these articles measured the binding affinity of nanobody-antigen complexes using SPR technology. We collected additional 114 complexes with the provided specific nanobody sequences and their corresponding affinity values. Some antigen sequences were retrieved from the PDB or UniProt databases.

We generated all possible pairwise combinations among the 185 samples to construct a sufficiently large dataset. In each group, if the first complex had a higher Kd value than the second (i.e., weaker binding strength), the group was labeled as positive. Using this balanced dataset, NanoBind-pair (Fig. 5A) was trained to predict the relative binding affinity between nanobody-antigen complexes.

To evaluate the performance of NanoBind-pair, we constructed three independent test sets with 100%, 50%, and 0% overlap with the training set. Specifically, 100% overlap means that all complexes in the test set have appeared in the training set but their pairwise combinations were not; 50% overlap indicates that half of the complexes in the test set were included in the training set but their pairwise combinations were not; and 0% overlap means that none of the complexes have appeared in the training set.

To establish an unbiased reference set for affinity estimation by NanoBind-affi, we selected 49 complexes with distinct Kd values from the initial pool of 185 nanobody-antigen pairs based on two strict criteria. First, the selected anchors must comprehensively cover the 10^{-12} to 10^{-4} M range, which encompasses the vast majority of nanobody-antigen affinities. Second, we maintained an approximately equal number of anchors per order of magnitude. Sorted by Kd from smallest to largest, these 49 anchors define 50 contiguous affinity ranges. This selection strategy ensures a uniform interval division across the binding energy landscape, while naturally providing finer resolution in the high-affinity (low Kd) region to facilitate the screening of high-potency candidates. Detailed information for the 49 complexes is provided in Supplementary Table S6.

Supplementary Note 5 | Homology-based evaluation demonstrates robust generalization

To further rigorously assess generalization, we constructed additional homology-based test subsets from the 0% split. Using CD-HIT[1], we partitioned the test set based on the antigen sequence identity relative to the training set at a 40% threshold. Specifically, we generated four subsets: Subset 1: Both antigens in the pair share <40% identity with the training antigens; Subset 2: One antigen shares <40% identity and the other >40%; Subset 3: One antigen shares <40% identity and the other is a 100% match; Subset 4: All remaining samples. Detailed test results are provided in Supplementary Tables S7 and S8.

We observed that performance decreased in the order of Subset 1, Subset 2, Subset 4, and Subset 3. This variance is primarily driven by the degree of structural divergence between the two target antigens within a comparison pair. In Subset 3, pairing a training-identical antigen with a highly novel one introduces massive structural discrepancy, complicating the relative ranking. Conversely, although both antigens in Subset 1 are completely novel to the model, they may share high sequence identity. This conserved structural context acts as a controlled variable, significantly simplifying the pairwise comparison. Importantly, in the downstream affinity estimation phase, the target complex is consistently compared against fixed reference anchors. Therefore, the practical application scenario most closely mirrors the conditions of Subset 3. Even under this specific scenario, the model retains robust generalization capability, achieving a reliable average F1-score of 0.648 and an MCC of 0.294.

Supplementary Note 6 | Structural and functional overview of the SARS-CoV-2 spike

The spike protein (S) of SARS-CoV-2, a homotrimer, plays a crucial role in viral entry by mediating interactions with host cell receptors, particularly ACE2. Each of the three polypeptide chains that form the spike protein contributes to its structure[2], with one receptor-binding domain (RBD) often adopting an "up" conformation to facilitate receptor binding. Several key sites within the spike protein have been implicated in viral infectivity and antibody neutralization. The RBD (PDB ID: 7OAY) contains critical residues, including Y489, F486, N487, and N501, which influence the virus's interaction with neutralizing antibodies and host receptors.

Understanding the structure and function of these critical sites in the spike protein, particularly the RBD, is vital for developing targeted therapeutics and vaccines aimed at neutralizing SARS-CoV-2. The identification of potent binding sites and their role in viral infectivity can aid in the design of future antiviral strategies.

The interface prediction results (Fig. 6A) showed that the antigen-interface residues identified by NanoBind-site highly overlapped with experimentally resolved data. Among the 22 residues predicted as binding residues by NanoBind-site, 20 residues were supported by experimental evidence. Two high-scoring predicted potential binding residues, V503 and S373, lack direct contact evidence but are mechanistically plausible. Specifically, the V503 residue is adjacent to the ACE2-binding epitope, and its hydrophobic side chain contributes to maintaining the stability of the RBD trimer interface. Under the background of the N501Y mutation, it may enhance receptor binding by forming additional hydrogen bonds. The S373 residue is positioned between interface residues A372 and F374, spatially sandwiched in the binding cleft, suggesting it forms part of a contiguous interaction surface and merits experimental validation as a functional contact.

Supplementary Note 7 | Molecular dynamics simulation for binding free energy calculation

In this study, the binding free energy between a nanobody and an antigen was calculated using the comprehensive binding free energy estimation software BFEE3. The computation was

based on the experimentally determined three-dimensional structure of the protein complex (PDB ID: 7OAY). The free energy was estimated via the geometric route method, with molecular dynamics (MD) simulations performed using the Gromacs engine. Simulations were conducted in the following computational environment: Gromacs 2024.3, Python 3.13, and BFEE (Binding Free Energy Estimator) v3.0.0.

For detailed instructions on the installation and usage of BFEE3 and CHARMM-GUI, please refer to Supplementary References[3,4]. This section provides an overview of the simulation workflow and parameter settings to facilitate the reproduction of our results (Supplementary Figure S2). The antigen protein was treated as the receptor and the nanobody protein as the ligand. Topology and coordinate files compatible with the MD engine were generated using CHARMM-GUI for both molecules. The system was solvated in a rectangular water box with a margin of 20.0 Å, and all other options were set to default.

Following system preparation, the BFEE3 plugin was employed to generate a complete simulation workflow for binding free energy calculation through the geometrical route. The workflow comprised several sequential stages executed in a Gromacs environment. It began with initial energy minimization and equilibration of the solvated complex. Subsequent stages involved conformational sampling in the bound state along the RMSD reaction coordinate, followed by systematic sampling of the ligand's orientational degrees of freedom relative to the receptor. This included sampling of three Euler angles (θ, φ, ψ) and two polar angles (θ, φ). The simulation then proceeded to sample the intermolecular distance coordinate, including a further minimization step, equilibration, and potential of mean force (PMF) calculation along the separation pathway. Finally, conformational sampling was performed in the unbound state along the RMSD coordinate. Each simulation stage was initiated only after the completion of the preceding stage, with all simulations conducted at a reaction temperature of 300 K.

The simulation times required for reasonable convergence were estimated based on runs performed on a computational system equipped with 64 CPU cores and a single NVIDIA A100 GPU. The overall computed binding free energy corresponds to a dissociation constant (Kd) of 3.662×10^{-10} M, which aligns well with the predicted affinity range of [3.2×10^{-10}, 4.1×10^{-10}] M.

Supplementary Note 8 | Intersection of NanoBind-seq and NanoBind-pro ensures high prediction precision

To evaluate the precision dynamics of NanoBind-seq and NanoBind-pro when applied individually across varying thresholds, we calculated their respective prediction precisions on the test set of the nanobody-antigen binding dataset. Then, we treated the simultaneous application of NanoBind-seq and NanoBind-pro as a virtual model, where the intersection of candidates exceeding the specified thresholds was defined as the predicted positive samples to calculate the prediction precision. As illustrated in Supplementary Figure S3, the precision within this intersection consistently improves as the threshold increases. Notably, when the prediction thresholds for both models are set to 0.57 and above, the intersection precision reaches exactly 1.0.

Supplementary Note 9 | Experimental details of the interpretability analysis

Here, we present the experimental procedures of the interpretability analysis using one representative complex, 9ETL, as an example. This case illustrates how each specific analysis was performed (see Supplementary Figures S4-S8 for results on other 4 samples).

In 9ETL, the nanobody chain (d) with three CDRs of lengths 7, 6, and 13 residues (annotated from the SAbDab-nano database), harboring 30 binding residues. The corresponding antigen chain (a) contains 34 binding residues.

For the Global Adaptive Module, for each CDR residue, we calculated the sum of the attention scores it received from the 26 CDR residues, defined as its total received attention weight. As a control, we randomly selected 26 non-CDR residues. For each non-CDR residue, we computed the sum of the attention scores it received from the 26 non-CDR residues, defined as its total received attention weight. We then compared the distributions of attention weights between the 26 CDR residues and 26 non-CDR residues using the Mann-Whitney U test (a non-parametric test for assessing differences in the central tendency of two independent samples). The result showed a significantly higher attention weight for CDR residues than for non-CDR residues (Fig. 7E and H, $p = 3.8 \times 10^{-6}$ and 7.2×10^{-6}), indicating that this module successfully allocates greater computational focus to the CDR regions, which

are critical for binding.

For the Local Adaptive Module, we sequentially masked (i.e., replaced with zero vectors) the embedding vector corresponding to each of the 26 CDR residues individually, and measured the resulting change in the predicted probability score. As a control, we performed the same masking procedure on 26 randomly selected non-CDR residues. The Mann-Whitney U test showed that masking CDR residues caused a significantly greater impact on the output score than masking non-CDR residues (Fig. 7F and I, $p = 4.3 \times 10^{-4}$ and 5.5×10^{-6}), confirming the module's specific reliance on CDR-derived features.

For both the Global Adaptive Module and Local Adaptive Module mentioned above, on the output of ESM-2, we sequentially masked (i.e., replaced with zero vectors) the embedding vector corresponding to each of the 26 CDR residues individually, and measured the resulting change in the predicted probability score. As a control, we performed the same masking procedure on 26 randomly selected non-CDR residues. The Mann-Whitney U test showed that masking CDR residues caused a significantly greater impact on the output score than masking non-CDR residues (Fig. 7G and J, $p = 2.0 \times 10^{-4}$ and 2.7×10^{-4}). This demonstrates that the model's decisions are causally dependent on CDR information, and the insensitivity to non-CDR masking further confirms that the model does not spuriously rely on framework regions.

The Co-Activation Module in NanoBind-seq is designed to model nonlinear binding synergy through element-wise (Hadamard) multiplication. Biologically, strong binding emerges only when complementary functional groups (e.g., H-bond donor/acceptor, hydrophobic patches, charged pairs) are precisely aligned—both partners must contribute. Hadamard multiplication enforces this cooperative logic: a high activation in any dimension occurs only if both sides contribute strong, matching signals, mirroring the all-or-nothing nature of residue-level complementarity. To validate that high-activation dimensions encode genuine synergistic complementarity, we sequentially masked (i.e., set to zero) the top 16 dimensions with the highest activation values in the feature vectors output by the Co-Activation Module and measured the corresponding changes in the predicted probability score. As a control, we performed the same masking procedure on equal number of dimensions randomly selected from the remaining ones. The Mann-Whitney U test showed

that masking high-activation dimensions had a significantly greater impact on the output score than masking random dimensions. We repeated the same experiment for the top 32 and top 64 highest-activation dimensions, observing consistent results (Fig. 7K-M, $p=3.1 \times 10^{-5}$, 7.4×10^{-10}, and 1.2×10^{-16}). This confirms that these dimensions capture the most discriminative interaction features, confirming these dimensions capture the most discriminative interaction features.

For the Cross-Assist Module in NanoBind-site, we computed cumulative attention weights from 30 known nanobody-binding residues to their 34 antigen-binding residues. The weight for each antigen-binding residue was defined as the sum of attention scores directed to it from all 30 nanobody-residues. As controls, we selected 30 nanobody non-binding residues randomly and computed their cumulative attention weights to 34 antigen random non-binding residues. Residues with weights below 0.01 were filtered out. The Mann-Whitney U test showed that the weight distribution for binding-residue groups was significantly higher than that for non-binding groups (Supplementary Figure S8A, $p=5.4 \times 10^{-3}$). This result proves that the module attends to true binding interfaces and captures interaction patterns.

Supplementary References

1. Huang, Y., Niu, B., Gao, Y., Fu, L. & Li, W. CD-HIT Suite: a web server for clustering and comparing biological sequences. *Bioinformatics* **26**, 680–682 (2010).
2. Wrapp, D. *et al.* Cryo-EM structure of the 2019-nCoV spike in the prefusion conformation. *Science* **367**, 1260–1263 (2020).
3. Fu, H. *et al.* Accurate determination of protein:ligand standard binding free energies from molecular dynamics simulations. *Nat. Protoc.* **17**, 1114–1141 (2022).
4. Fu, H., Chipot, C., Shao, X. & Cai, W. Achieving Accurate Standard Protein–Protein Binding Free Energy Calculations through the Geometrical Route and Ergodic Sampling. *J. Chem. Inf. Model.* **63**, 2512–2519 (2023).
